# Lipid self-assembly dependence on hyaluronic acid size reveals biolubrication and osteoarthritic degeneration mechanisms

**DOI:** 10.1126/sciadv.adz9517

**Published:** 2026-01-14

**Authors:** Kangdi Sun, Mark W. Rutland, Rosa M. Espinosa-Marzal

**Affiliations:** ^1^ Materials Science and Engineering Department, The Grainger College of Engineering, University of Illinois at Urbana-Champaign, Urbana, IL 61801, USA.; ^2^ Department of Chemistry, KTH Royal Institute of Technology, Stockholm SE-100 44, Sweden.; ^3^ School of Chemistry, University of New South Wales, Sydney 2052, Australia.; ^4^ Laboratoire de Tribologie et Dynamique des Systèmes, École Centrale de Lyon, Lyon 69130, France.; ^5^ Bioeconomy and Health, Materials and Surface Design, RISE Research Institutes of Sweden, Stockholm 114 28, Sweden.; ^6^ Civil and Environmental Engineering Department, The Grainger College of Engineering, University of Illinois at Urbana-Champaign, Urbana, IL 61801, USA.

## Abstract

Hyaluronic acid (HA) and phospholipids (PLs) are key components of joint lubrication. In osteoarthritis (OA), the molecular weight (MW) of HA is reduced, which has been proposed to weaken the anchoring capacity of PL and impair lubrication. This study reveals a different mechanism by directly linking the MW to the structure of HA-PL (hybrid) assemblies and frictional properties. Using mixed-MW HA and PL to model this difference between healthy and OA synovial composition, we found interfacial lamellar structures form under healthy-like conditions, while hybrid vesicles predominate in OA-like conditions. At physiologically relevant shear rates, lamellar assemblies maintain ultralow friction, whereas vesicles are removed, causing a tenfold friction increase. These findings provide mechanistic insight into how HA-PL structural organization controls lubrication. While this simplified system does not capture the biochemical complexity of synovial fluid, this study advances understanding and offers a framework for designing structure-informed therapeutic strategies and biomimetic lubricants.

## Teaser

Hyaluronic acid (HA) with phospholipids forms lamellar films that resist shear, while osteoarthritic HA forms fragile vesicles.

## INTRODUCTION

Nature has developed water-based lubrication systems that significantly outperform most human-made devices. In the hip joint, where articular cartilage is subjected to compressive pressure exceeding 5 MPa, the friction coefficient (CoF) can be as low as μ ∼ 0.001 (*1*, *2*). Numerous experimental and theoretical investigations have been conducted to better understand the effective mechanisms behind joint lubrication. Hydration lubrication (*3*), a mechanism first proposed by Klein and co-workers, has emerged as a key concept in understanding the ultralow friction observed in articular joints (*4*–*6*). This mechanism relies on the idea that water bound to surface charges remains fluid at the interface between two sliding surfaces, even under high pressures, effectively serving as a highly efficient water-based lubricant.

Synovial joints are complex systems. The aqueous synovial fluid contains phospholipids (PLs), hyaluronic acid (HA), and lubricin, among other components that adsorb on the surface of hyaline cartilage, a specialized connective tissue composed of collagen type II, proteoglycans and a small amount of chondrocytes, the resident cells (*7*). PLs have been proposed to play a critical role in hydration lubrication of articular joints, since they self-assemble into well-ordered structures such as vesicles and lamellae, stacked lipid bilayers, that strongly bind water molecules (*8*–*10*). Several studies have compared the lubrication performance of vesicles and lamellar structures (*11*, *12*). For instance, the work by Sorkin *et al.* (*12*) suggested that vesicles exhibit superior lubrication properties under relatively high pressure due to their ability to form stable, closed structures that dissipate shear stress more effectively than lamellar structures.

HA was initially thought to also play an important role in reducing friction in synovial joints (*13*, *14*). More recent studies have challenged this view (*8*, *9*, *15*, *16*). Using a surface force balance (SFB), Klein and collaborators demonstrated that HA is a poor boundary lubricant under physiological conditions, because it fails to form stable boundary films. This finding aligns well with earlier works, suggesting that HA primarily contributes to increasing the viscosity of the synovial fluid (*17*–*19*), rather than directly reducing friction at the molecular level. Subsequently, focus shifted toward understanding the interactions and co-assembly between HA and PLs (*20*, *21*) and their synergistic effects in boundary lubrication (*20*, *22*, *23*). Klein and collaborators proposed that HA is anchored at the outer surface of articular cartilage by lubricin, and complexes with phosphatidylcholine, a phospholipid, to provide efficient hydration lubrication (*9*). Conversely, Dėdinaitė *et al.* (*4*) reported no synergistic effects beyond the enhanced adsorption of vesicles when HA was preadsorbed on the surface.

The composition of HA in the synovial fluid has been reported to significantly change during the development of osteoarthritis. For a healthy joint, HA exhibits a molecular weight (MW) of 2 to 20 MDa with a concentration ranging from 1 to 4 mg/ml. In osteoarthritic joints, not only is the HA broken down, resulting in lower MW, but its concentration is also reduced by a factor of 10 (*24*, *25*). Recently, we used small-angle neutron scattering (SANS) to examine the self-organization of HA and PL in the fluid with either high- or low-MW HA. A combination of microbalance measurements and nanoscale imaging revealed that high-MW HA promotes the formation of thicker films, suggesting their higher load-bearing capacity (*21*). This work interrogates the relation between structure and lubrication of boundary films consisting of PL and mixtures of low- and high-MW HA to simulate this compositional difference of the synovial fluid between healthy and osteoarthritic conditions. Previous studies with HA anchored to gelatin surfaces suggested that PL vesicles are more easily removed from a surface coated with low-MW HA compared to high-MW HA, which led to an increase in CoF (*22*). Our novel approach, nanoscale friction visualization, reveals a different mechanism and thus resolves previous controversies about the synergistic effects between HA and PL, in both simulated healthy and osteoarthritic conditions.

## RESULTS

For this study, dipalmitoylphosphatidylcholine (DPPC) was selected because it is among the most abundant PLs in synovial fluid (*26*) and has a reproducible phase behavior (*27*). While simplistic, using a single, well-characterized PL minimizes variables related to acyl chain unsaturation or oxidation (*28*) and allows us to isolate the fundamental physicochemical effects of the interactions with HA. Unilamellar DPPC vesicles were prepared by thin film hydration followed by the standard extrusion method and diluted to 0.2 mg/ml in 150 mM NaCl. DPPC mixtures of HA with different MWs are more biomimetic than single MW HA (*29*, *30*). Therefore, HA with MWs of 30 to 50 kDa (low-MW HA) and 100 to 1000 kDa (high-MW HA) was added at 1:2 and 2:1 (low MW:high MW) mass ratios to the PL-vesicle solution and incubated for at least a day before any testing. The 1:2 ratio reflects high-MW HA–enriched formulations that are often considered in viscosupplementation therapies (*31*), and we chose 2:1 ratio to represent the relative depletion of high-MW HA observed in osteoarthritic joints. The two model systems were designed to resemble only this compositional difference between healthy and OA synovial fluid and are subsequently labeled as (simulated) healthy and OA conditions.

Since both PL and HA are present in the synovial fluid (*19*, *32*) and their interaction is strong (*33*, *34*), we adopt a different sample preparation to previous works and allow a biomimetic boundary film formation by adsorption from solution, rather than attaching HA to the surface before the adsorption of vesicles. HA and vesicles self-assemble into hybrid vesicles in the solution with an average diameter larger than that of the PL vesicles: 150 ± 60 nm (healthy), 137 ± 55 nm (OA) versus 127 ± 33 nm (pure liposomes); see results from dynamic light scattering (DLS) in fig. S1 in the Supplementary Materials. Details of the sample preparation and methods can be found in Materials and Methods.

The choice of gold as the model substrate enables direct correlation of the adsorbed masses from quartz crystal microbalance (QCM), with the film nanostructure and the spatial distribution of the friction force measured by atomic force microscopy (AFM). While a gold surface does not resemble the composition of the cartilage surface, this experimental approach eliminates the need for arbitrary assumptions regarding the binding of the boundary films as they self-assemble on the surface. Notably, the wettability of the gold substrate (fig. S2 and table S1) before exposure to the hybrid vesicles is remarkably similar to that of a delipidized cartilage surface (*35*). Hence, we contend that the similar wettability of gold and cartilage surface can lead to similar boundary films compositions. Figures S3 to S5 display QCM data illustrating the formation of the films and analysis details. While the average thickness of the boundary films formed under healthy and OA conditions is comparable (77.0 ± 8.9 nm versus 96.5 ± 11.7 nm), the stiffness under simulated healthy conditions is much greater (10.4 ± 1.55 kPa versus 3.2 ± 1.49 kPa), suggesting that they are more robust. In contrast, reference QCM experiments involving only HA mixtures (no PL) reveal the formation of very thin films (3 to 4 nm) without any significant difference between the two compositions (fig. S3, D and E). This also suggests that precoating with HA is unlikely to influence the adsorption of PL, giving further support to the biomimetic adsorption approach used here.

**Fig. 1.**
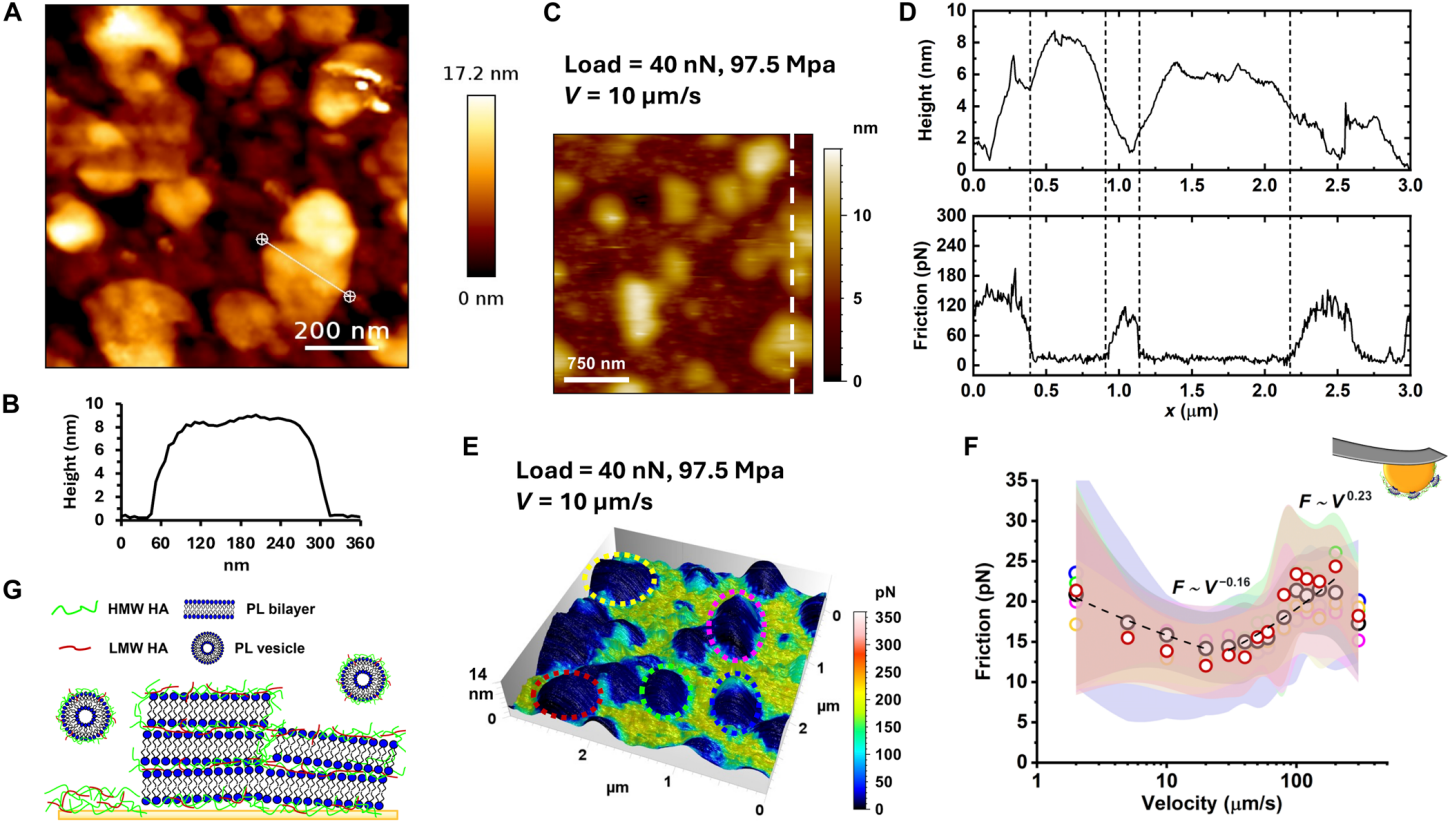
Boundary films and lubrication under simulated healthy condition at 37°C. (**A**) Topographic image of the surface film measured by QI AFM and (**B**) cross-section height profile across a lamellar region. In QI, the tip approaches, contacts, and retracts from the sample to generate a force-distance curve at each pixel of the image. This is done repeatedly across the entire image grid, giving a full topographic/mechanical profile of the sample. The height image shows a height difference of ~8 to 10 nm, about 2 to 2.5× the reported thickness of a DPPC bilayer (*63*–*65*); see details of analysis in fig. S6. (**C**) Topographic image taken while the tip applies a load of 40 nN and slides over the surface at 10 μm/s. The cross-section along the dashed line and the corresponding friction force are shown in (**D**). (**E**) Friction image superposed to topographic image under the same conditions as in (C) to (D). (**F**) Friction versus velocity at various locations in (E); see color code. (**G**) Representation of the surface film upon adsorption of the hybrid vesicles. Five different samples were prepared and investigated by AFM and QCM. AFM QI images (topographic measurements) were collected on a minimum of five different locations per sample. On each image, at least five representative lamellae were identified and analyzed. AFM friction force images were carried out on a minimum of five lamellae per image (five images per sample). Friction histograms were extracted from each vesicle and a friction average and SD were obtained for each of them and are shown in (F). The shadows in (F) represent the standard deviation (SD) of each histogram. More details can be found in the “Statistical analysis” section. Note that HA/PL is present in the solution during AFM measurements and films adsorb also on the gold microsphere.

Figure 1A displays the biomimetically surface-assembled structure under simulated healthy condition. Quantitative imaging (QI) by AFM is particularly well-suited for soft materials, as it minimizes deformation. Surface features were segmented on the basis of height thresholds and curvature to differentiate structural components; see details of the analysis in fig. S6. The analysis reveals relatively flat domains with a surface coverage of ~37.7%, which indicates that healthy HA-PL leads to the formation of lamellar structures on the surface. Representative cross-section height profiles of lamellae are shown in Fig. 1B and fig. S7. Note that AFM typically measures a relative height difference of ~8 to 16 nm (though it can reach up to 40 nm) on the flat domains, whereas the total film thickness determined by QCM is more than 70 nm. There are various contributions that explain this difference, most importantly, AFM does not measure the total film thickness but only the surface topography, and hence, height differences. Furthermore, HA molecules extending into solution will also contribute to the QCM thickness but not be imaged by the AFM tip; and the film thickness measured (weighted) by QCM includes the bound water.

**Fig. 2.**
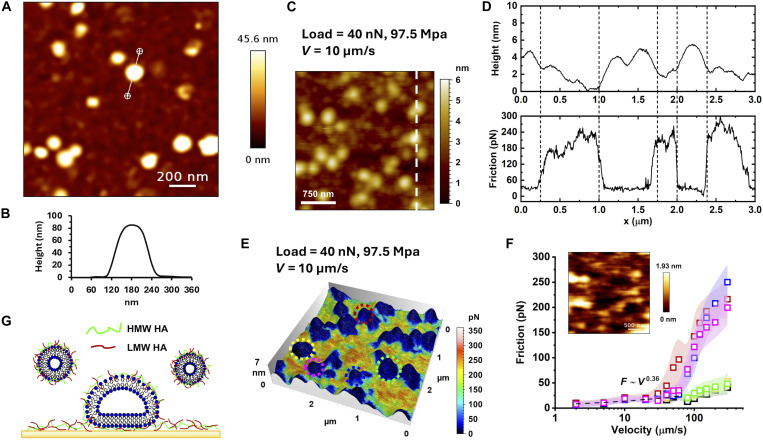
Boundary films and lubrication under simulated OA condition at 37°C. (**A**) Topographic image of the surface film and (**B**) Cross section along a vesicle, a round cap-like shape with height ~85 nm (**C**) Topographic image taken while the tip applies a load of 40 nN and slides over the surface at 10 μm/s. The cross section along the dashed line and the corresponding friction force are shown in (**D**). (**E**) Friction image superposed to topographic image under the same conditions as in (C to D). (**F**) Friction versus velocity at various locations in (E); see color code. (**G**) Representation of the surface film upon adsorption of the hybrid vesicles. Three different samples were prepared and investigated by AFM. AFM QI images (topographic measurements) were collected on a minimum of five different locations per sample. On each image, at least five representative vesicles were identified and analyzed. AFM friction force images were carried out on a minimum of five vesicles per image (five images per sample). Friction histograms were extracted from each vesicle and a friction average and SD was obtained for each of them and are shown in (F). The shadows in (F) represent the SD of each histogram. More details can be found in the “Statistical analysis” section. Note that HA/PL is present in the solution during AFM measurements and films adsorb also on the gold microsphere.

Upon adsorption of OA HA-PL complexes, a very different film structure is observed (Fig. 2A). Notably, there is a greater proportion of vesicle-like structures with their characteristic, distinctly rounded, cap-like morphology and ~80 nm in height, as illustrated by the cross-section height profiles in Fig. 2B. The surface coverage with vesicles is significantly reduced (12.61%; fig. S6). Thus, vesicles retain their spherical integrity rather than spreading into lamellar structures, suggesting that the differences in HA-lipid interactions and self-assembly results in enhanced fusion tendency of the larger MW hybrid vesicles. This distinct film structure explains the different viscoelastic behavior of the two films, as vesicles are softer and more deformable than lamellar structures. Note that our imaging approach (QI) allows us to unambiguously distinguish between vesicles and lamellar structures since the low imaging forces preclude substantial deformation. Previous studies (*9*, *12*, *20*) proposed that AFM imaging in tapping mode led to the marked deformation of vesicles to a height between 3 and 8 nm. Our observations indicate that some of these structures could rather have been bilayers or lamellae.

Vesicle rupture is primarily driven by strong interactions with the substrate and adjacent lamellar structures (*36*–*38*). Upon adsorption to a surface, the vesicle deforms due to adhesive interactions, with the most pronounced curvature occurring slightly above the surface near the edge of the adsorbed region. This region of high curvature is energetically unfavorable (*39*). In our prior work, we observed that high-MW HA adsorbs onto the surface of dispersed DPPC vesicles, enhancing the interaction between hybrid vesicles and the surface (*21*). DLS and SANS proved that the high-MW HA results in a compressed bilayer and smaller hybrid vesicles, further increasing curvature and instability at the edge of the adsorbed bilayer region. This may explain the easier rupture of hybrid vesicles compared to those formed with low-MW HA. Given the higher proportion of high-MW HA under simulated healthy conditions, the increased prevalence of lamellar structures and greater surface coverage can be attributed to enhanced interfacial interactions between hybrid vesicles and the surface. Vesicle rupture also induces rupture of neighboring vesicles (*40*), leading to the formation of lamellar patches, as interpreted from the topography images.

A gold microsphere (~2.7 μm in radius) glued to a tipless AFM cantilever was used to apply a load over the surfaces while sliding at constant velocity in reciprocal motion (trace and retrace) to measure friction by AFM in contact mode (*41*, *42*). Both sphere and surface were immersed in an aqueous saline solution with HA-PL complexes simulating either healthy or OA conditions, so that the boundary films self-assembled on the surfaces of both the substrate and the microsphere. Although the curvature has been proved to be a factor influencing the self-assembly structure of PL (*43*–*45*), the microsphere is ~30 to 250× greater than the characteristic thickness of the surface-adsorbed HA-PL films. At this scale, the local surface can be regarded as quasi-planar, and thus the packing and orientation of the HA-lipid assemblies are not expected to differ significantly. In contact mode, the AFM generates two topographic images (trace and retrace) while measuring friction in situ, enabling an unambiguous relation between the friction force and the HA-PL nanostructure to be obtained. We refer to this approach as friction visualization. Figure 1C displays an in situ image of the boundary film during such friction measurements for the simulated healthy condition, and Fig. 1D shows the corresponding height and friction force along the dashed line; more friction images can be found in fig. S8. The friction force is significantly smaller over the lamellar structures than on the surrounding regions. The observed tilt in the height profiles may be due to the deformation of the lamellar structures, arising from their compliant nature. Note that much higher forces are applied during friction measurements compared to those applied during QI imaging (Fig. 1, A and B).

Figure 1F displays the results of velocity-dependent friction measurements (2 to 300 μm/s) using the colloid probe directly on individual lamellar aggregates. Joints typically operate at 100 μm/s to 10 mm/s, covering similar and higher velocity ranges. The marker colors correspond to measurements on different lamellar structures, as indicated in Fig. 1E, which shows the overlaid images of topography and friction; contact pressures are shown in table S2. Initially, friction decreases with velocity reaching a minimum at ~20 μm/s. This small decrease in friction typically indicates the action of weak yet attractive interactions at the sliding interface that are rate dependent (*46*); therefore, as the velocity increases, the adhesive bonds between the surfaces or films have insufficient time to form, leading to a reduction in friction. Above this speed, friction begins to increase with velocity as $F\propto V^{n}$ (*n* ~ 0.23) up to ~200 μm/s, and then slightly decreases. This power-law relation between friction and velocity for boundary (surface-adsorbed) films is typically attributed to viscous dissipation upon shear of the interface, which here exhibits a shear thinning behavior, since the exponent is smaller than 1. This means that friction not only results from the shear of water, as when an oil lubricates an engine, but also from the shear of the surface-adsorbed HA-PL films. This has been also reported for polymer brushes and denoted as thin film lubrication (*47*, *48*). In situ height images reveal that the decrease in friction at very high velocity occurs when the colloid partially lifts from the surface, transitioning to a lubrication regime dominated by the solution; see fig. S8.

Figure 2C displays the in situ topography of the boundary film during friction force measurements at 40 nN and 10 μm/s for the OA condition; other friction images can be found in fig. S9. The boundary film is once again rougher and more heterogeneous than that self-assembled under healthy conditions even under applied load. Instead of the lamellar structures shown in Fig. 1C, features with higher local curvature and size consistent with the diameter of the vesicles in solution are imaged on the surface. Figure 2D shows the cross section along the dashed line and the corresponding friction force. The average vesicle height decreases from ~45 to ~6 nm during friction-force measurements, indicating that the vesicles undergo substantial deformation and flattening as a result of the high applied loads. The friction force between colloid probe and vesicles remains relatively low under low loads and slow speeds, and it gradually increases with velocity, $F\propto V^{n}$ (*n* = 0.36), again dominated by the viscous dissipation in shearing the boundary film. However, as the sliding velocity surpasses ~60 μm/s, a drastic and abrupt increase in friction is observed, exceeding the values measured under healthy conditions. In situ images reveal that this transition coincides with partial removal of the boundary film; the inset in Fig. 2F shows an example of partial removal. Once structural disruption of some vesicles occurs, neither the vesicular morphology nor the low-friction state recovers within the experimental timescale, indicating that the deformation (rupture) or removal and friction increase are irreversible. The large variation of the friction force observed at high velocities (Fig. 2F) can be attributed to the vesicle size distribution. Differences in composition, nanostructure and dimensions likely lead to variations in the ease of disruption and removal, and consequently, in friction (see DLS in fig. S1).

**Fig. 3.**
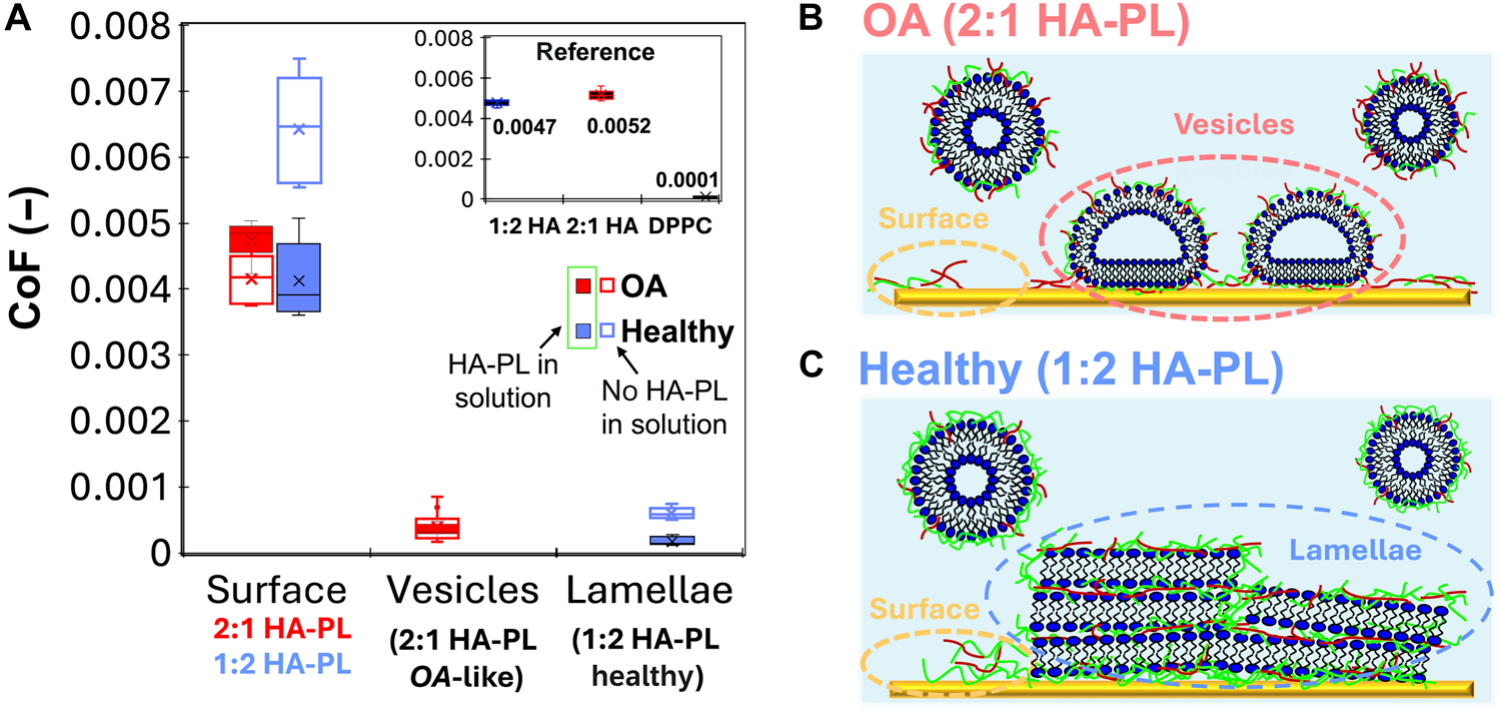
CoFs of boundary films for healthy-like and OA-like conditions and reference systems at 37°C. (**A**) CoF summary. Friction was measured on lamellar structures, vesicles and surrounding surface using a microsphere in function of load. Filled boxes (healing possible) refer to biomimetic conditions, where the HA-PL complexes are present in fluid and can re-adsorb on substrate and microsphere surfaces. CoFs were also determined after the adsorption of the hybrid complexes on the two countersurfaces, but in the pure saline solution to hinder self-healing (unfilled boxes ). Inset shows the CoFs from reference measurements with only DPPC and only HA mixture in the aqueous solution (no complexes). All measurements were carried out at a constant sliding velocity of 5 μm/s. Friction images and friction versus load scatter plots are displayed in figs. S10 to S16. Friction force measurements were carried out on at least five different regions per image and sample. CoFs were determined from friction versus load scatter plots on each region by linear fitting. More details can be found in the “Statistical analysis” section. (**B** and **C**) Schematic illustration of the interfacial structures and friction measurement configurations under OA-like (B) and healthy-like (C) conditions. (B) Under OA-like conditions, hybrid vesicles dominate the interface. The adsorbed layer mainly consists of flattened hybrid vesicles (red dashed circle), while the yellow dashed circle marks the surrounding surface region, which may be partially covered by HA or remain bare. (C) Under healthy-like conditions, stacked HA-PL lamellae form at the interface (blue dashed circle), providing a continuous boundary film. The yellow dashed circle indicates the adjacent surface region, with or without HA coverage.

The lubricious behavior of biomimetic systems is typically reported in terms of a CoF. Figure 3 (full boxes) presents the CoF determined as the slope of friction versus load at a constant sliding velocity of 5 μm/s. Corresponding friction images as function of applied load are shown in figs. S10 and S11 and average friction versus load plots in figs. S12 and S13. The measurements reveal a low CoF for both healthy and OA systems, but measurably higher for vesicles (OA) versus lamellae (healthy) (2.8.10^−4^ ± 3.6.10^−5^ versus 1.7.10^−4^ ± 6.42.10^−5^), consistent with Figs. 1 and 2 at 5 μm/s.

In all experiments described so far, HA-PL complexes were present in the aqueous solution, so that readsorption onto the surfaces (self-healing) is possible if material removal occurs, biomimetically simulating the conditions in articular joints. The so-called self-healing of boundary films refers to their ability to re-adsorb or reform after being disrupted by shear. Whether this happens depends strongly on sliding velocity. If shear is slow (long contact time), molecules have sufficient time to diffuse, reorient, and readsorb within the contact area, so the film remains continuous and lubricious and friction stays low. If shear is fast, the time is too short for the film to reform, and friction increases.

To examine the role of self-healing of the boundary films, load-dependent friction measurements were also carried out in the absence of HA-PL complexes in solution. This prevents self-healing from happening and can be compared to previous results. Figure 3 (unfilled boxes) shows that the CoF is now smaller on vesicles (OA) than on lamellar (healthy) structures (2.5.10^−4^ ± 1.2.10^−4^ versus 7.3.10^−4^ ± 1.1.10^−4^), but much more scattered; see also figs. S14A and S15A. The lamellar structures thus have a slightly elevated CoF in the absence of solution complexes. It is not immediately clear that this minor difference is related to the absence of self-healing effects; if so, it would appear to be a small effect, possibly due to dissolved large MW HA being drawn into the contact and anchoring lamellar structures, which otherwise appear to be impervious to damage. It is also likely that any PL hydrophobic tail regions exposed (for example, due to stripping of a PL leaf during sliding) with commensurately higher energy, can be cloaked by hydrophobic adsorption from the solution phase, either by HA or indeed PL material.

On the other hand, velocity-dependent friction measurements in the absence of solution complexes reveal that, once again, vesicles are removed at high velocities, leading to a drastic increase in friction; figs. S14C and S15C. Compared to the case of HA-PL complexes in solution, it is clear that there is no self-healing possible at high sliding speeds, and the damage is irreversible. In contrast, for the lamellar structures, friction remains small across the entire investigated range of sliding velocities, supporting the idea that there is no damage of the lamellar structure, and hence, no healing of the boundary film is necessary.

Figure 3 also shows that the CoF on lamellae and vesicles are much lower than the CoF provided by the surrounding surfaces (labeled as “surface”), which may be coated by HA, PL, or their mixture; see also figs. S14 and S15. The CoF of these regions is of the same order as the reference measurements for HA (inset in Fig. 3 and fig. S16, B and C), which suggests that the surface is coated with a film composed solely of HA [1:2 and 2:1 low:high-MW HA show similar CoFs (4–6.10^−3^)]. In comparison, pure PL vesicles alone provide extremely low (velocity-independent) friction, as proposed for hydration lubrication. Thus, in agreement with literature (*49*, *50*), HA alone is a poor lubricant, at least in comparison to DPPC.

## DISCUSSION

In conclusion, this study provides insights into molecular mechanisms underlying boundary lubrication in a model system consisting of HA and DPPC, with the potential implication for understanding osteoarthritis pathology. Distinct structures and lubrication behaviors were observed in the two synovial fluid–simulated conditions, underscoring the importance of the molecular weight of HA and its specific interaction with DPPC; see cartoons in Figs. 1G and 2G.

A higher proportion of low-MW HA (simulated OA condition) results in more vesicles than lamellar structures in the boundary films and increased friction at higher velocities due to their relatively easy removal. The selected range of velocities (2 to 300 μm/s), although smaller than the slip velocities observed in joints (walking, 0.1 to 1 mm/s; running, ~1 to 10 mm/s), is nonetheless higher than in key pioneering studies (*5*, *10*, *51*). Moreover, our approach allows for a reasonable extrapolation of the results to higher velocities and shows that self-healing of hybrid vesicles after removal is not possible. Previous works invoked a weaker attachment of the PL to the surface by low-MW HA through an anchoring process. We show instead that the lower resistance of the OA boundary film is actually due to the fact that the predominating hybrid vesicles are intrinsically less well attached. The robustness under healthy conditions is rather due to a completely different morphology of the adsorbed PL structure, namely, the lamellar structure that appears to be preferred in the presence of high-MW HA. In the presence of the complexes in solution, the lamellar structures also display a lower coefficient of friction over a wide range of sliding speeds.

While previous studies revealed a velocity-independent low friction for vesicles (a marker of hydration lubrication), our measurements show a small velocity dependence under all conditions. We believe that this discrepancy is mainly due to the higher applied pressures by AFM (61.41 to 105.2 MPa) compared to SFB measurements [<22 MPa (*52*)]. The pressure used in AFM experiments is deliberately higher than the average values in articular joints (walking, ~3 to 6 MPa; running, ~6 to 10 MPa; jumping, >15 MPa). Higher pressures are expected at asperity contacts (*53*, *54*) and in OA pathological conditions, where cartilage degradation leads to elevated and uneven pressures during activities like walking, running, and high-impact movements. The surface roughness is indeed also more pronounced when vesicles are more abundant on the surface (in the simulated OA condition). This also hinders the transition to full fluid lubrication, increases localized contact stresses, and exacerbates wear. These findings highlight the importance of considering surface smoothness and roughness in simulating articular joint lubrication.

Notably, our work also demonstrates that vesicles do not always exhibit superior lubricity compared to lamellae, in contradiction with literature (*12*, *55*). Rather the reverse appears to be true under biomimetic conditions, underscoring the relevance of synergistic effects. We show here that vesicles can undergo marked compression to thicknesses where they could be confused with lamellae. It is conceivable that this fact has earlier led to misinterpretation and that lamellar structures have been interpreted as compressed vesicles due to the large deformations that tapping mode can induce. The in situ QI imaging used here is not subject to this issue and provides higher-resolution topography images, enabling a clear distinction between lamellar and vesicular structures. In addition, friction imaging directly provides a link between the structure of the boundary films and the lubrication behavior in situ. Although it has been proposed (*5*, *12*), perhaps counterintuitively, that pure PL vesicles are more robust and stably attached to the substrate than pure PL lamellar structures, the opposite is observed here. High-MW HA chains apparently reduce the exposure of thermodynamically unfavorable hydrophobic tail boundaries of the lamellar structures, maintaining robustness and lower friction.

It must be emphasized, however, that the present system is intentionally, and necessarily, simplified and does not include the full range of molecular constituents found in native synovial fluid, such as glycoproteins, sulfated glycosaminoglycans, or link proteins, all of which could potentially influence film structure and function to some degree. Likewise, the “healthy” and “osteoarthritic” conditions simulated here should be viewed as qualitative representations based on HA MW only; in vivo, joint disease involves additional factors including altered enzyme and MMP activity, oxidative environments, and pH variations. In addition, inflammatory lipids such as unsaturated phosphatidylcholine, species, fatty acids, or oxylipins may also alter interfacial structure and lubrication. Synergistic effects of other joint components are thus possible, indeed likely, but remain to be investigated. Future studies should aim to incorporate even more realistic systems to further expand upon these findings. Overall, this work advances our understanding of lubrication mechanisms in healthy and OA-affected joints, providing a foundation for developing targeted therapeutic strategies to mitigate friction and wear in pathological conditions.

## MATERIALS AND METHODS

### Preparation of DPPC small unilamellar vesicles and HA mixtures

Small unilamellar vesicles (SUVs) were prepared by thin film hydration followed by the extrusion method (*56*). In brief, a solution of DPPC in chloroform was transferred to a round-bottom borosilicate flask and the solvent was evaporated via rotary evaporation. The dry lipid thin film was kept under vacuum overnight to ensure complete solvent removal. The lipids were rehydrated by adding NaCl solution (in 150 mM NaCl in H_2_O) to get DPPC stock solution (1 mg/ml) under nitrogen purging to avoid lipid contact with oxygen. The resuspended lipids were vortexed for 10 min to disperse the lipids in the solution. The resulting solution was extruded at least 23 times at 47°C (above the DPPC phase transition temperature ~44°C), each time through two polycarbonate membranes with a pore size of 0.2 and 0.1 μm, respectively, to form unilamellar vesicles with a narrow size distribution.

Two kinds of HA stock solutions of 1 mg/ml were made by dissolving the HA in the NaCl solution at 4°C for 48 hours with continuous stirring to ensure complete dissolution. The stock solutions were diluted to a final concentration of 0.5 mg/ml and mixed with each other in different ratios to form 0.5 mg/ml low:high 1:2 HA (healthy) and low:high 2:1 HA (OA) stock solutions. Then, the HA mixtures were mixed with DPPC SUV (0.2 mg/ml) and incubated for at least 1 day before any testing. Reference DPPC SUV and reference HA mixtures (1:2 and 2:1) stock solutions were prepared by the same processes as described above.

The pH of the healthy simulated condition was 5.93 ± 0.02, and that of the OA-like condition was 5.87 ± 0.02. The pH of the pure solvent (water with 150 mM NaCl) was 6.02 ± 0.02. The same solution batches were used for QCM-D, DLS, and AFM measurements, with pH recorded both immediately after preparation and after the experiments (drift <0.05 pH units). No buffering agents were added to prevent possible specific adsorption of buffer ions onto the gold surface or lipid headgroups. Figure S18 demonstrates that changes in pH in the range 5.87 to 7.3 do not affect the self-assembly of HA and PL in solution.

### Dynamic light scattering

Bulk size distribution was measured using a Malvern Zetasizer Nano ZS two-angle particle and molecular size analyzer (Malvern Instruments, Worcestershire, UK). The light source is a He-Ne laser at 633 nm, and the temperature was set at 37°C. Disposable folded capillary cells (DTS1070) were used for DLS measurements. Each sample was measured in triplicate. The Malvern Zetasizer software (version 7.02) was used to analyze the obtained data. All DLS data were plotted according to the intensity distribution of the hydrodynamic diameter (*D*_h_).

### Quartz crystal microbalance

AT-cut quartz crystals (diameter = 25 mm) coated with gold (QSX 301, LOT Oriel Group, Germany) with a fundamental frequency of 4.95 MHz were used to study the surface adsorption and structural transitions of the HA-DPPC mixtures in 150 mM NaCl in Millipore water. Notably, these gold surfaces have similar wettability as delipidized cartilage, as the contact angle is ∼50° (table S1) for gold sensors versus 39° and 56° (after 21- and 3-min delipidized treatment) for cartilage (*35*). Furthermore, gold surfaces are smooth enough (RMS roughness ∼1.26 nm, see fig. S19) to enable us to image the adsorbed films immersed in solution by AFM and distinguish the film morphology, which is impaired on rough surfaces.

Changes in frequency (Δ*f*) and dissipation (Δ*D*) of the crystal resonator (Q-Sense, Sweden) were measured over different overtones (from *n* = 3 to 11), but the results presented here are at overtones of 5, 7, 9, and 11, which are adequate for the thickness of the adsorbed films (tens of nanometers). Gold sensors were cleaned by ultraviolet (UV) ozone for 10 min, immersed in a solution 5:1:1 mixture of MilliQ water:ammonia (25%): peroxide hydrogen (30%) at 75°C for 5 min, rinsed with Milli-Q water, dried with N_2_, and again treated with UV ozone for 10 min before use. Inlet and outlet tubing and the QCM chambers were rinsed with ultrapure water (GenPure UV, TKA GmbH, Niederelbert, Germany) before use. The fundamental frequencies were characterized in the NaCl solution. The chamber is designed to provide a nonperturbing exchange of liquids over the quartz crystal by means of a pump. A flow rate of 150 μl/min was used, and the chamber temperature was maintained at 37°C during all measurements. The average film thickness and viscoelastic properties of adsorbed HA-PL complexes after rinsing with solution were analyzed using the Voigt viscoelastic model (*57*) with the QSense DFind software. The details of model and analysis are described in fig. S5.

### Atomic force microscopy

After adsorption, the QCM sensors with the adsorbed films were imaged by AFM (Nano Wizard, JPK Instruments, Germany) using the QI mode with a sharp tip (HQ:CSC37, no Al, 0.3 to 0.8 N/m, NanoAndMore, USA) at 37°C. This method creates maps of height (or topography), adhesion, and surface stiffness. QI is the chosen mode for AFM imaging, since it eliminates the application of a lateral force as it occurs in contact mode imaging, and thereby, it minimizes artifacts due to drag or damage, which can happen to soft films. Although tapping mode also reduces drag and damage, QI mode more effectively images soft polymeric interfaces in liquid environments by adjusting the set point force to detect the interface. QI images (10 × 10 μm^2^, 5 × 5 μm^2^, and 2 × 2 μm^2^) were postprocessed using the JPK software by subtracting a polynomial fit and replacing empty pixels by interpolation. The height profiles were extracted using Mountains software [Mountains SPIP Academic 9, Surface analysis software version 9.2 (64-bit), Digital Surf, France] to identify and distinguish the components (vesicles or lamellae). The caption of fig. S6 explains the analysis method in detail.

Spherical smooth gold particles with a radius ~2.7 μm were produced by electrical discharge between gold wires (0.25 mm) under an argon atmosphere (*58*). The gold colloidal tip was prepared by gluing the gold sphere to a tipless cantilever (HQ:CSC37, tipless, no Al, 0.3 to 0.8 N/m, NanoAndMore, USA). The sphere radius and roughness were determined before and after measurements (to determine if wear or irreversible deformation happened) by reverse imaging a sharp grating (TGTZ-400, TED PELLA Inc); see fig. S20. Friction maps were generated using contact mode imaging by AFM to visualize the frictional and topographic characteristics of the surface films. During contact mode AFM imaging of the sensor surface, the tip slides in forward and reverse direction along a scan line at constant load and velocity before moving to the next scan line. Contact mode imaging produces height images during trace and retrace of the tip. Because of the friction force between tip and sensor surface, the tip deflects in the lateral direction, and with the lateral spring constant of the cantilever, the lateral force during trace and retrace can be determined. The friction image results from half the difference of the lateral deflection in forward and reverse directions (*59*).

Load-dependence friction measurements were conducted in 150 mM NaCl solution at constant velocity of 5 μms^−1^ and loads from 10 to 50 nN. The velocity-dependent friction measurements were conducted in 150 mM NaCl solution under a constant normal load of 40 nN and a sliding velocity from 2 to 300 μm s^−1^. HA-PL complexes were present in the solutions unless otherwise indicated. The friction data obtained was processed using Mountains software. For both healthy and OA condition, at least five different regions were selected for the analysis of each feature (vesicles, surface, and lamellae). Friction histograms for each region were exported as text files, and the average friction and SDs were determined and shown in Figs. 1 to 3 and in the Supplementary Materials.

The contact pressures were estimated by using Hertz model for a sphere-on-flat configuration, for a gold microsphere against a flat gold sensor in liquid. The adhesion is negligible in force-distance curves in liquid, so a non-adhesive Hertz model is appropriate. The contact radius *a* was determined from

$$a={\left(\frac{3\mathrm{FR}}{4E^{*}}\right)}^{1/3}\;\text{with}\;\frac{1}{E^{*}}=\frac{1-\nu_{1}^{2}}{E_{1}}+\frac{1-\nu_{2}^{2}}{E_{2}}$$

Where *F* is the applied normal load, *R* is the probe radius, and E* is the effective modulus. Using typical values for gold (*E* ≈ 78.6 GPa and ν ≈ 0.42), the mean contact pressure [$\bar{p}=F/\left(\pi a^{2}\right)$] falls in the range ~61 to 105 MPa (table S2). The Hertz model can be applied if *a*/*R* (*a* is the contact radius) and *d*/*h* (*d* is the indentation depth from $a=\sqrt{\mathrm{Rd}}$ and *h* the gold coating film thickness ~100 nm) ratios are smaller than 0.3 and 0.1, respectively. This is fulfilled here, indicating small deformation with no substrate effects (*60*). This estimation neglects the influence of the adsorbed HA-PL films, and hence, it is an overestimation. Note that a larger microsphere would lower further the nominal pressure at the expense of increasing the contact area, which would reduce imaging resolution of nanometer-thick films. To (i) maximize imaging resolution, (ii) reduce hydrodynamic artifacts, and (iii) test film robustness under worst-case scenario, microspheres with ~2.5 μm in radius were selected for this study.

### Statistical analysis

DLS, QCM, AFM QI imaging, and nanoscale-friction visualization measurements were performed on triplicate samples, at least. Specifically, five different samples were investigated for the healthy simulated condition, and three different samples for the OA simulated condition. We carried out two different QCM measurements with each sample (*N* = 10 and 6, for simulated healthy and OA condition, respectively). Three DLS measurements with each sample (*N* = 15 and 9, respectively). Five regions (areas) were imaged by AFM on each sample (*N* = 25 and 15, respectively) at three different scales for each region, and one friction measurement on each sample (*N* = 5 and 3, respectively) at multiple velocities and loads. Reference measurements included two different samples, each for 1:2 HA and 2:1 HA, and one sample for DPPC, and two experiments per sample were conducted by DLS, QCM, and AFM. DPPC has been comprehensively investigated in the literature and our results from DLS, QCM, and AFM are in good agreement with literature (*21*, *61*, *62*). For DLS measurements, error bars represent the SD of the size distribution. Representative QCM data are presented as scatter plots. The analysis of the QCM data led to values for the film thickness, elastic modulus, and film viscosity, which are reported as average values and SDs. AFM QI maps were collected at a minimum of five different positions per sample at scan sizes of 5 μm × 5 μm, 3 μm × 3 μm, or 1.5 μm × 1.5 μm. Cross-sectional height profiles of lamellae or vesicles were extracted from multiple locations across these maps. At least five representative lamellae or vesicles from different images were identified and analyzed under both healthy and OA-simulated conditions. AFM friction data were processed using Mountains software. For each condition (healthy and OA), a minimum of five distinct regions per sample were selected to analyze each surface feature (vesicles, lamellae, and bare surface). Friction histograms from each region were exported as text files and average friction values along with SDs were calculated using Microsoft Excel. CoFs under different conditions and surface features were determined by linear fitting of the data points and summarized with SDs (error bars), as shown in the box and whisker plot.

## Supplementary Materials

### This PDF file includes:

Figs. S1 to S20 Tables S1 and S2 References
