# Supplementary material for: Lipid self-assembly dependence on hyaluronic acid size reveals biolubrication and osteoarthritic degeneration mechanisms
Source: Sci Adv. 2026 Jan 14;12(3):eadz9517. doi: 10.1126/sciadv.adz9517 (PMC12802837; doi:10.1126/sciadv.adz9517)
Supplement: Supplementary file 1 — Figs. S1 to S20 Tables S1 and S2 References [file sciadv.adz9517_sm.pdf]

Science Advances
AAAS

Supplementary Materials for

Lipid self-assembly dependence on hyaluronic acid size reveals biolubrication and osteoarthritic degeneration mechanisms

Kangdi Sun *et al.*

Corresponding author: Rosa M. Espinosa-Marzal, rosae@illinois.edu

Sci. Adv. **12**, eadz9517 (2026)
DOI: 10.1126/sciadv.adz9517

This PDF file includes:

Figs. S1 to S20
Tables S1 and S2
References

Supplementary Figures

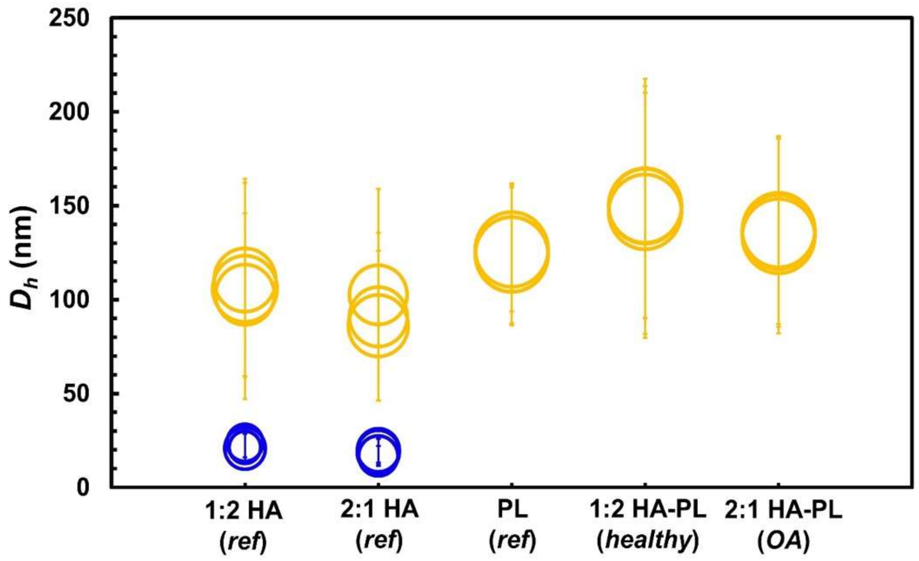

Fig. S1.
Size of HA/PL systems determined by Dynamic Light Scattering (DLS). Bubble diagram with hydrodynamic diameter 1:2 and 2:1 HA mixtures (no lipid added) and pure DPPC vesicles (PL); as well as 1:2 HA-PL (healthy) and 2:1 HA-PL (OA) complexes in solution. Each bubble shows the average radius of each peak and the error bars give the standard deviation of the distribution. The size of the bubble indicates the % contribution of each peak to the overall measured intensity. The reference HA samples (1:2 and 2:1) exhibit a bimodal size distribution in 150 mM NaCl, with smaller peaks at 19.34 nm and 21.83 nm (blue), and larger peaks at 90.88 nm and 105.7 nm (yellow) respectively. In contrast, pure DPPC vesicles displayed a unimodal size distribution with an average diameter of 127 nm. Upon mixing HA with DPPC vesicles, the resulting HA-PL complexes show also a unimodal distribution, with average diameters of 137 ± 55 nm under OA simulated conditions and 150 ± 60 under healthy simulated conditions. The plot shows the results for three different samples per condition, which cause the superposition of three bubbles.

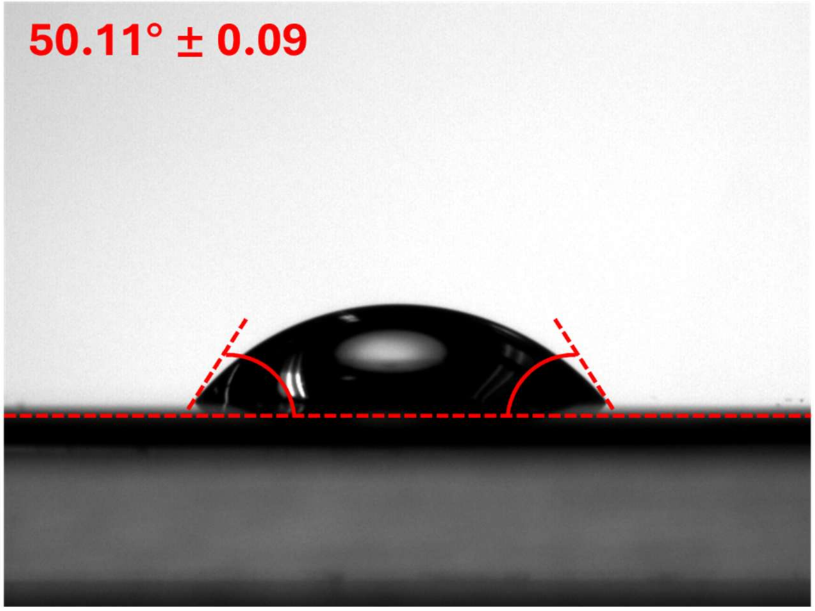

Fig. S2.
Water contact angle on a clean gold sensor used for QI and AFM measurements. The average contact angle (both sides) is 50.11 ± 0.09º in this example The results from other ten experiments are shown in **Table S1**.

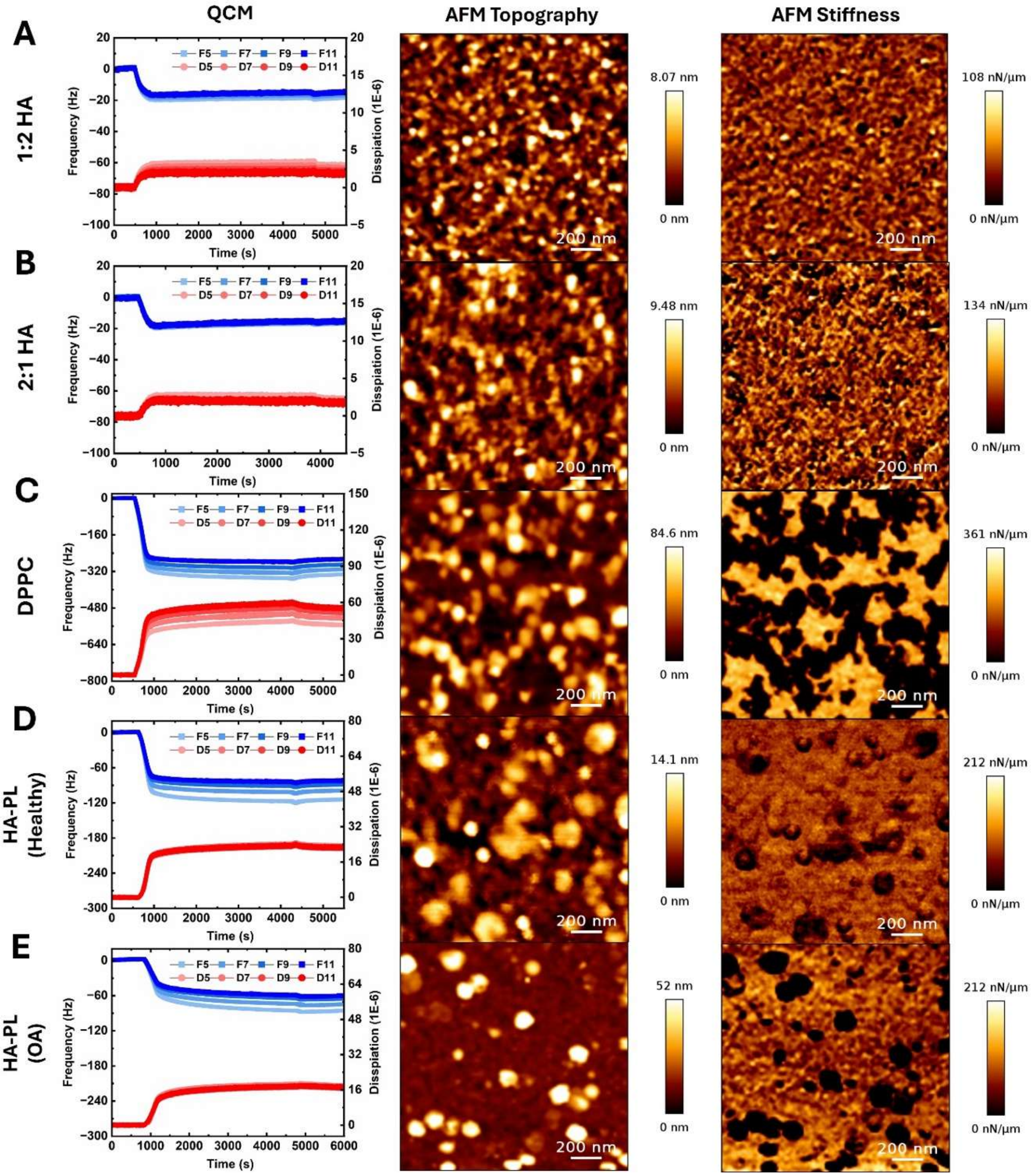

Fig. S3.

Representative QCM data and QI images after QCM measurements. (A) 1:2 HA and (**B**) 2:1 HA, **(C)** pure vesicles, **(D)** HA-PL complexes (healthy condition) and **(E)** HA-PL complexes (OA condition). Pure DPPC vesicles exhibit the highest frequency shift, indicating the greatest mass adsorption onto the surface. The adsorption of the reference HA mixtures (no DPPC, (A-B) leads to the smallest frequency shift. In comparison, HA-PL complexes show intermediate changes in both frequency and dissipation, suggesting reduced and possibly more heterogeneous adsorption compared to DPPC only. The adsorption process appears to involve competition between free HA molecules and HA-PL complexes. Under healthy conditions, the surface-adsorbed structures are stiffer, and flatter compared to the vesicular structures on the surface formed under simulated OA conditions.

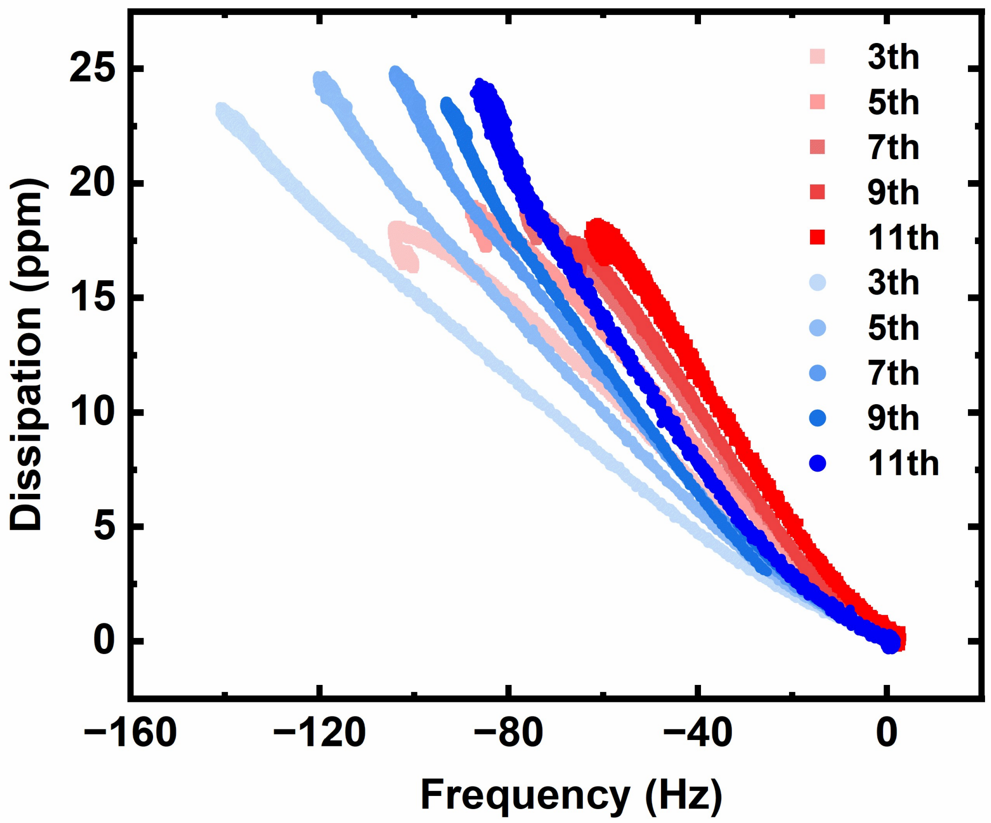

Fig. S4.

Dissipation vs. frequency plot corresponding to the adsorption of healthy and OA HA–PL complexes on gold surfaces. The slope of the dissipation–frequency curve is notably higher for the HA-PL mixtures under OA simulated conditions (red), indicating a more viscoelastic behavior of the adsorbed *OA* HA–PL complexes compared to that of *healthy* HA–PL complexes (blue).

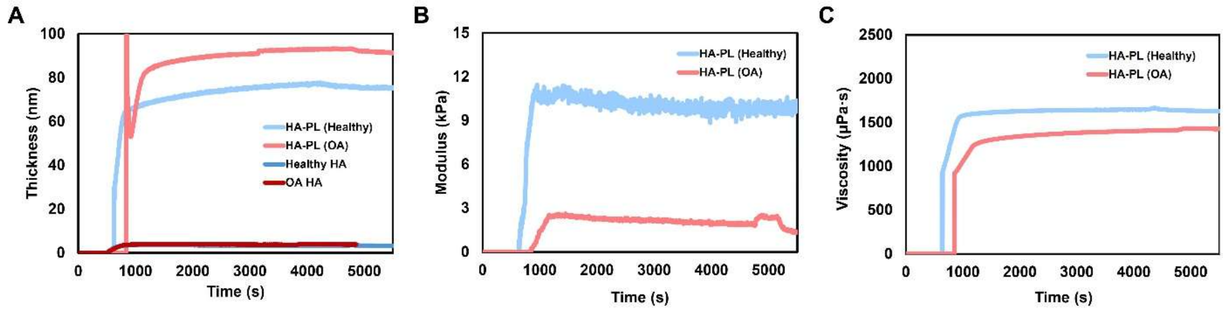

Fig. S5.

Thickness, modulus and viscosity of adsorbed films. The plot compares results for HA-PL (healthy) and HA-PL (OA) samples, i.e. 1:2 low MW to high MW HA, and 2:1 low MW to high MW HA. Raw frequency and dissipation data were fitted using a viscoelastic (VE) model to extract three key film properties: **(A)** thickness (h), **(B)** elastic shear modulus (G'), and **(C)** viscosity (η_f) (*57, 66*). The film thickness and viscoelastic parameters were obtained using the *SmartFit* routine in Dfind (QSense, Biolin Scientific), which performs a multi-overtone least-squares fit to the finite-thickness Voigt viscoelastic model (Kelvin–Voigt) for a homogeneous viscoelastic film coupled to a semi-infinite liquid phase (*57, 66, 67*). This was necessary for the HA-PL complexes, since they exhibit non-negligible dissipation, a footprint for viscoelasticity. *SmartFit* traces a physically smooth evolution of parameters in time by constraining the solution space and rejecting discontinuous solutions. The algorithm performs a multi-overtone Levenberg–Marquardt minimization (typically n=3rd–9th) to extract h, G', and η_f simultaneously, minimizing the global residual between measured and modeled Δf_n and ΔD_n.(*68*) In our analysis, the density and viscosity of the liquid phase (ρ_ℓ, η_ℓ) with PL-HA complexes was fixed to the experimentally measured values, η_ℓ =0.9 $\pm$ 0.018 mPa.s and 1.08 $\pm$ 0.016 mPa.s for OA- and healthy-simulated conditions, respectively. The density of the solutions (ρ_l) were calculated by harmonic mixing rule for density $\rho_{mix} = \sum_i w_i / \sum_i (w_i/\rho_i)$ and it is ρ_ℓ~1000 g/ml for both conditions as the water content is >99 wt.% in both solutions. The film density (ρ_f) was assumed to be 1050 kg/m^3, typical for dense packed hydrated polysaccharide/lipid assemblies (*69*). The final results reported in the manuscript are the values *after rinsing with the NaCl solution* (to ensure that the loosely bound material is not erroneously included*)*. Hence, the values of density and viscosity of the solutions with HA and lipids do not affect the final values, but only the values of the NaCl solution. The figure also shows the results for reference HA mixtures (1:2 and 2:1, no PL). In this case, the Sauerbrey equation was used to estimate the film thickness. This model assumes a rigid, uniform, and thin film with negligible viscoelastic contributions (negligible dissipation). Note that due to partial surface coverage and surface roughness, the extracted film thickness may not be fully accurate, as the VE model assumes a uniformly covered and smooth film.

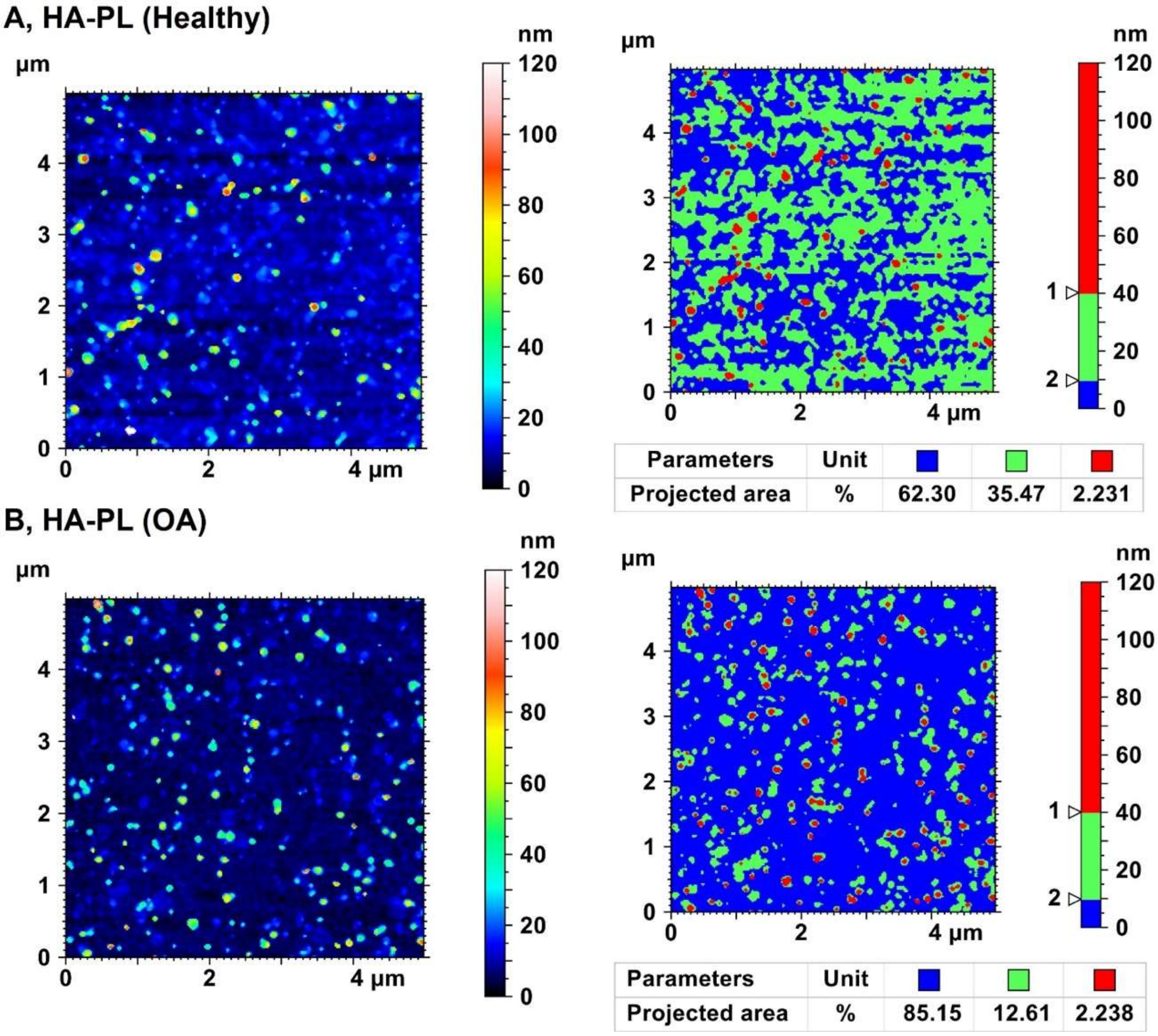

Parameters	Unit			
Projected area	%	62.30	35.47	2.231

Parameters	Unit			
Projected area	%	85.15	12.61	2.238

Fig. S6.

Topography images and surface coverage analysis of HA–PL films. Measurements on **(A)** *healthy* and **(B)** *OA*-simulated conditions. The analysis of the surface coverage was based on pixel-by-pixel height distributions obtained from multiple 3D topography images (at least three per composition). To differentiate distinct structural features, we defined height thresholds based on the characteristic profiles observed across all images. Planar, extended regions with lateral continuity and low curvature (see, e.g., **Fig. 1B** and **Fig. S7**) were identified as lamellae, whereas highly curved, circular domains exceeding 40 nm in height were classified as vesicles (**Fig. 2B** and **Fig. S7**). The lamellar structures consistently exhibited heights in the range of 8–40 nm, whereas vesicular features exceeded 40 nm and displayed rounded, cap-like cross-sectional shapes. Accordingly, we set a lower threshold of 8 nm to distinguish regions covered by HA molecules from the bare gold surface, and a second threshold of 40 nm to separate lamellar (8–40 nm) from vesicular (> 40 nm) domains. While we cannot exclude the possible presence of lipid monolayers or HA thin films with heights below 8 nm, the present analysis specifically targets mesoscale features (lamellae and vesicles) contributing to the boundary film morphology.

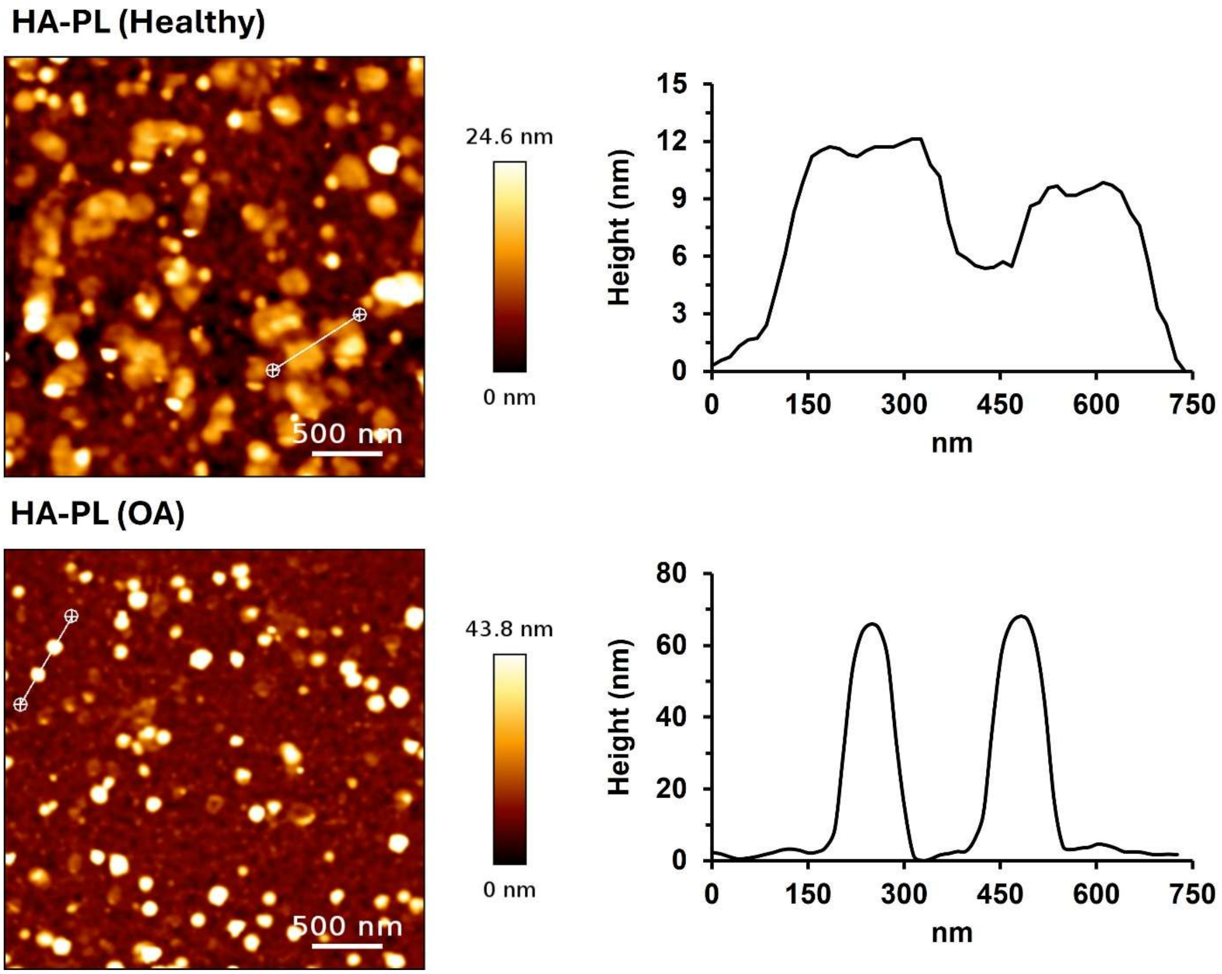

Fig. S7.
Additional topography images of the adsorbed films measured by AFM in QI mode. Top: HA–PL under healthy-like conditions. Bottom: HA–PL under OA-like simulated conditions. The plots on the right show cross-sections along the white lines in the topography images, highlighting the flat and rounded morphologies characteristic of adsorbed lamellae and vesicles, respectively.

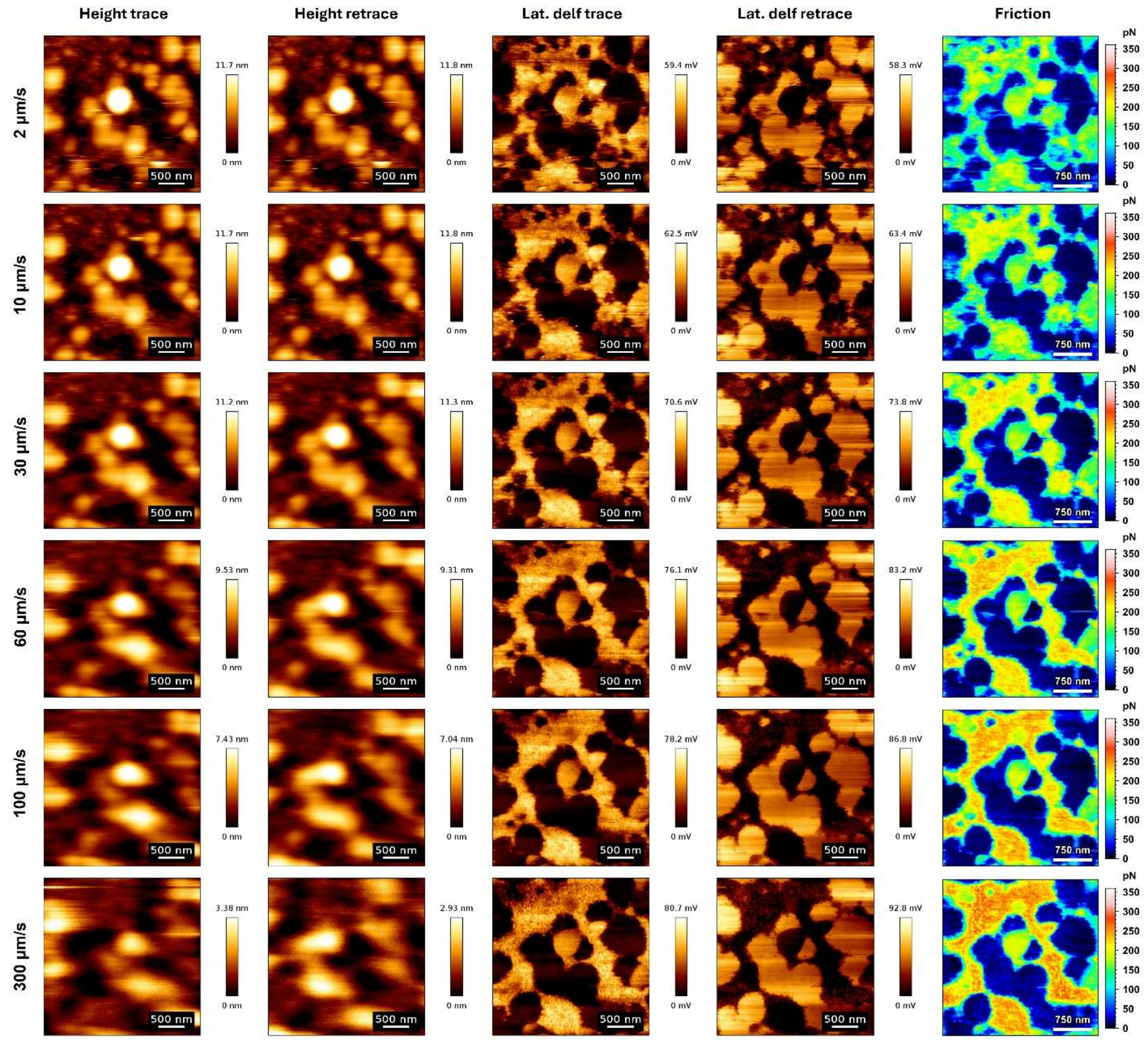

Fig. S8.

Trace and retrace height and lateral deflection images, and calculated friction images for *healthy* HA-PL films in the presence of complexes in the liquid phase. Applied load = 40 nN at sliding velocities of 2, 10, 30, 60 and 100 and 300 μm/s.

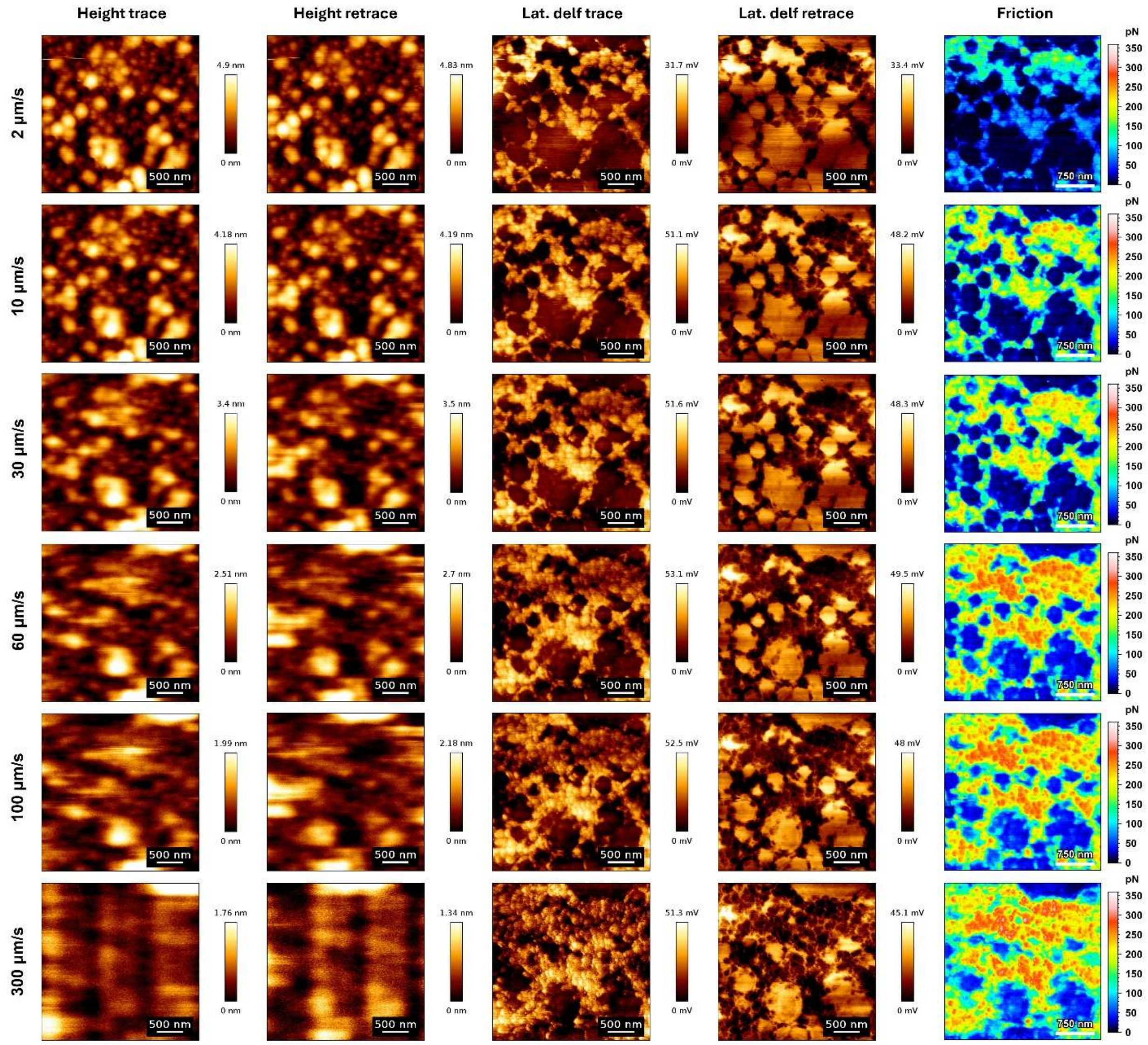

Fig. S9.

Trace and retrace height and lateral deflection images, and calculated friction images for *OA* HA-PL films in the presence of complexes in the liquid phase. Applied load = 40 nN at sliding velocities of 2, 10, 30, 60 and 100 and 300 µm/s.

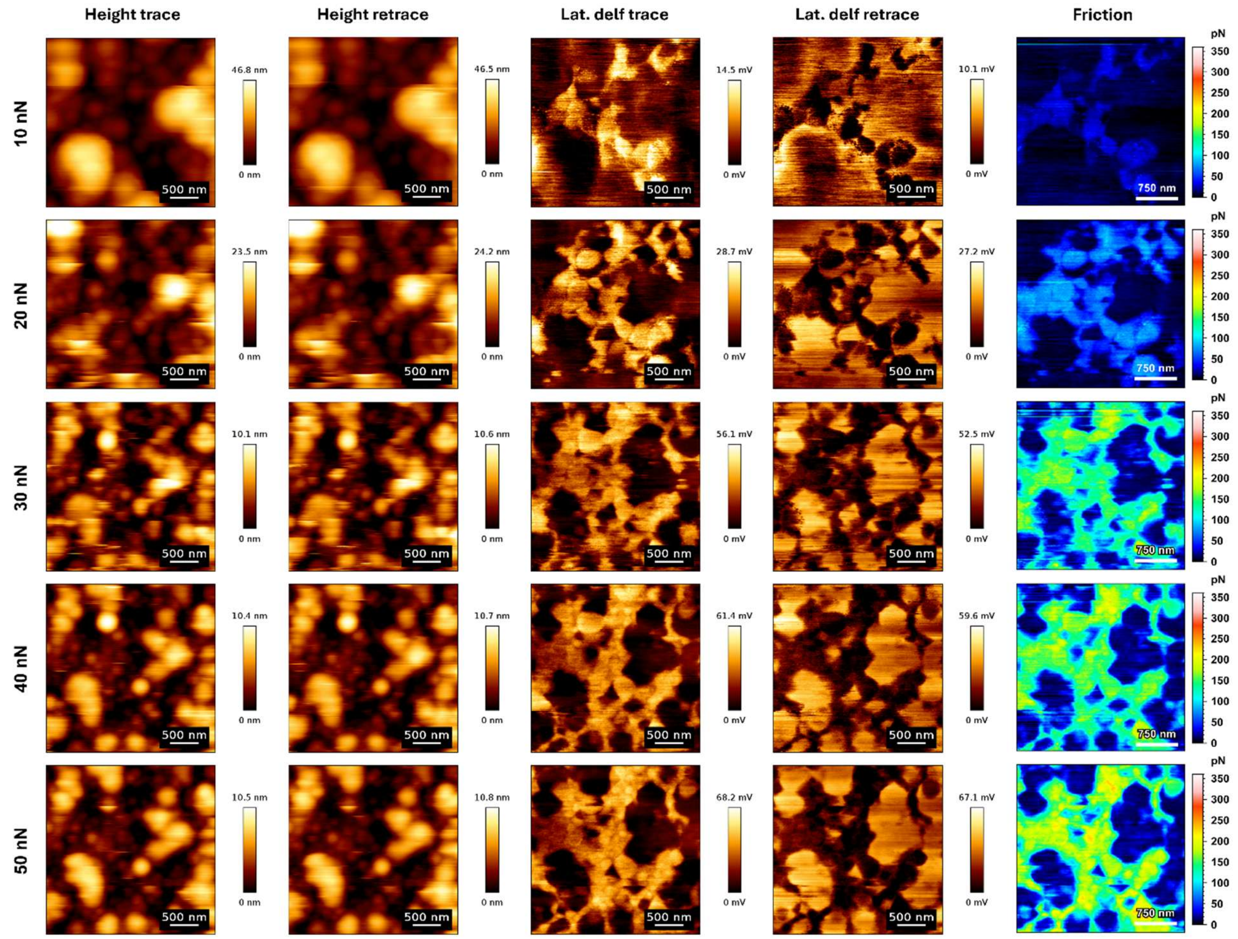

Fig. S10.
Trace and retrace height and lateral deflection images, as well as calculated friction images for *healthy* HA-PL films in the presence of complexes in the liquid phase. Sliding velocity = 5 μm/s under normal loads of 10, 20, 30, 40 and 50 nN.

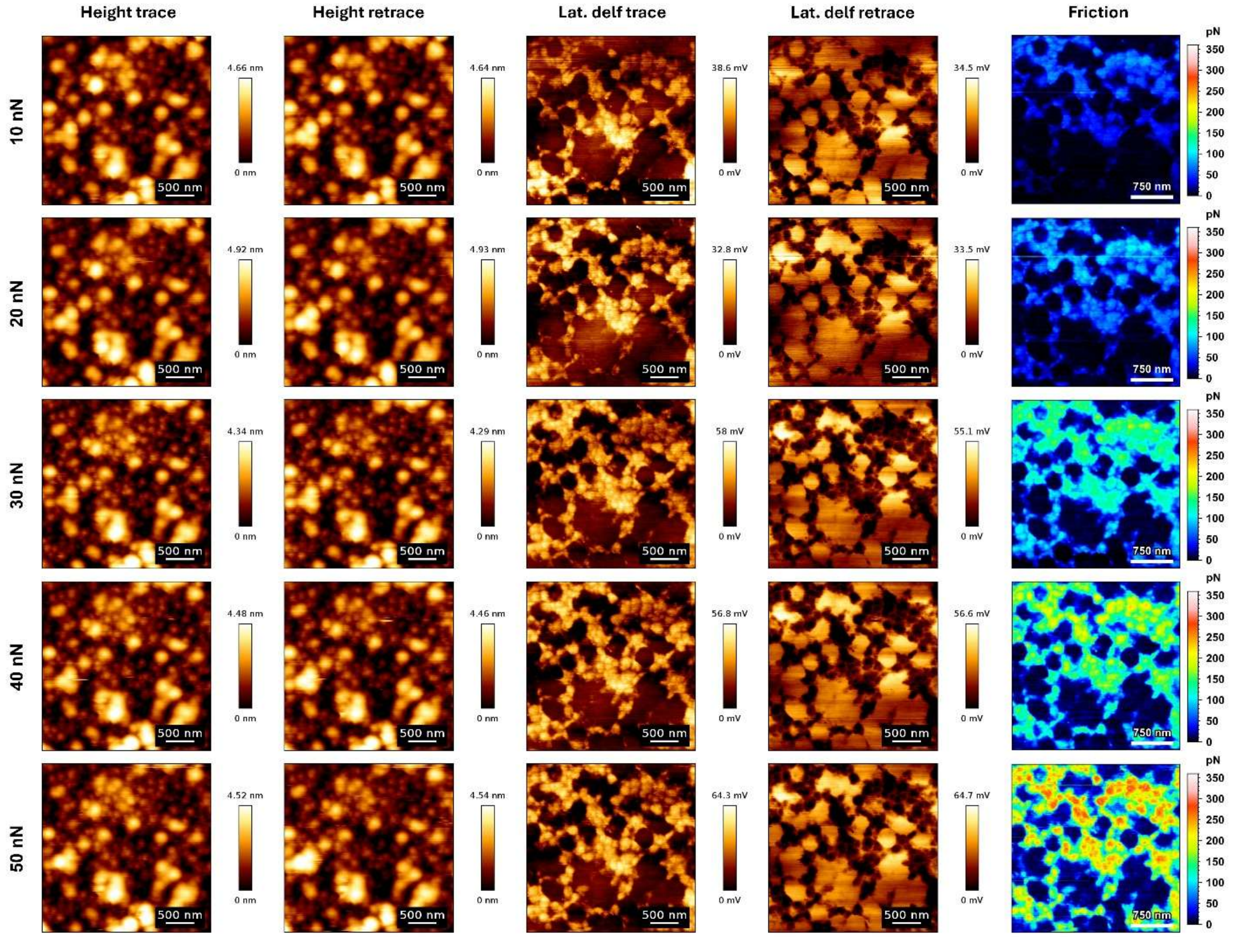

Fig. S11.
Trace and retrace height and lateral deflection images, as well as calculated friction images for *OA* HA-PL films in the presence of complexes in the liquid phase. Sliding velocity = 5 μm/s under normal loads of 10, 20, 30, 40 and 50 nN.

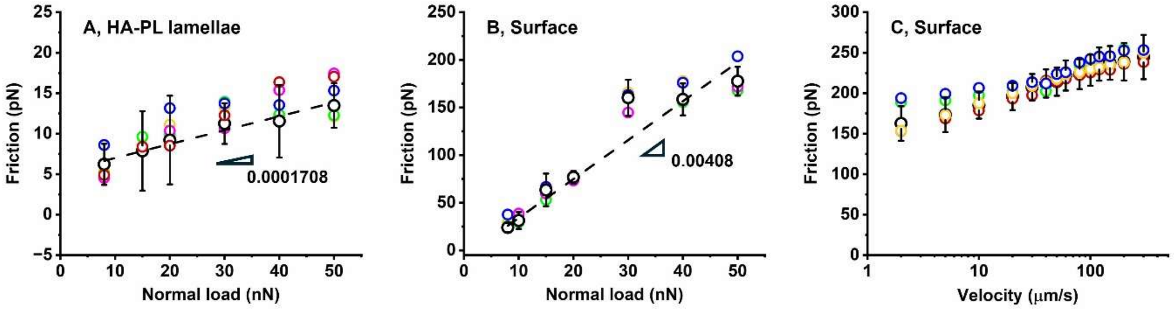

Fig. S12.

Friction measurements on *healthy* HA-PL films. Friction measurements were carried out with a gold colloid. Each color represents a spot on the surface: friction vs. load on (**A**) HA-PL lamellae and (**B**) surface, as well as (**C**) friction vs. velocity on the surface. Friction pixel values of all friction images were exported to histograms. Each data point represents the average friction and standard deviation of the friction histogram. Load-dependent friction measurements were performed at a fixed lateral sliding velocity of 5 μm/s. Friction vs. velocity was varied out under a constant normal load of 40 nN and sliding velocity ranging from 2 to 300 μm/s. These results suggest that the "surface" is coated with a film composed solely of HA, since 1:2 low:high MW HA show similar CoFs (0.004-0.006); see **Figs. S16B** and **S17C**. Each data point in the scatter plot represents the average of a friction histogram taken within a region of an image. The error bar represents the standard deviation of each histogram.

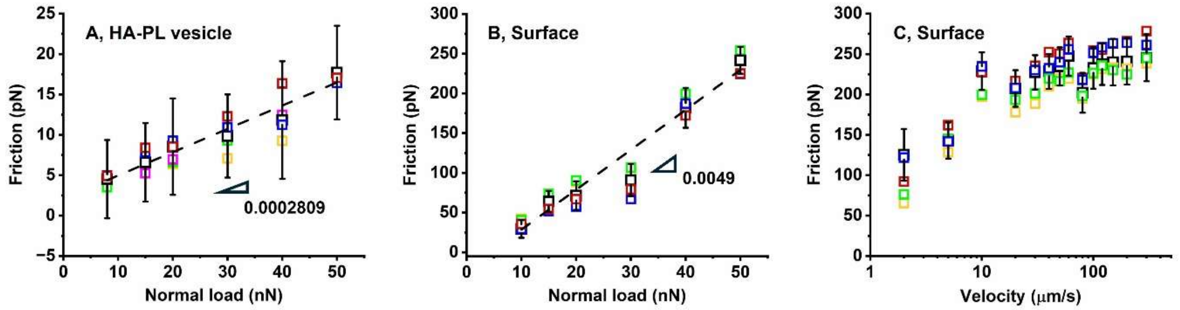

Fig. S13.

Friction measurements on *OA* HA-PL films. Friction measurements were carried out with a gold colloid. Each color represents a spot on the surface: friction vs. load on (**A**) HA-PL vesicles and (**B**) surface, as well as (**C**) friction vs. velocity on the surface. Friction pixel values of all friction images were exported to histograms. Each data point represents the average friction and standard deviation of the friction histogram. Load-dependent friction measurements were performed at a fixed lateral sliding velocity of 5 μm/s. Friction vs. velocity was varied out under a constant normal load of 40 nN and sliding velocity ranging from 2 to 300 μm/s. These results suggest that the "surface" is coated with a film composed solely of HA, since 2:1 low:high MW HA show similar CoFs (0.004-0.006); see **Figures S16C** and **S17C**. Each data point in the scatter plot represents the average of a friction histogram taken within a region of an image. The error bar represents the standard deviation of each histogram.

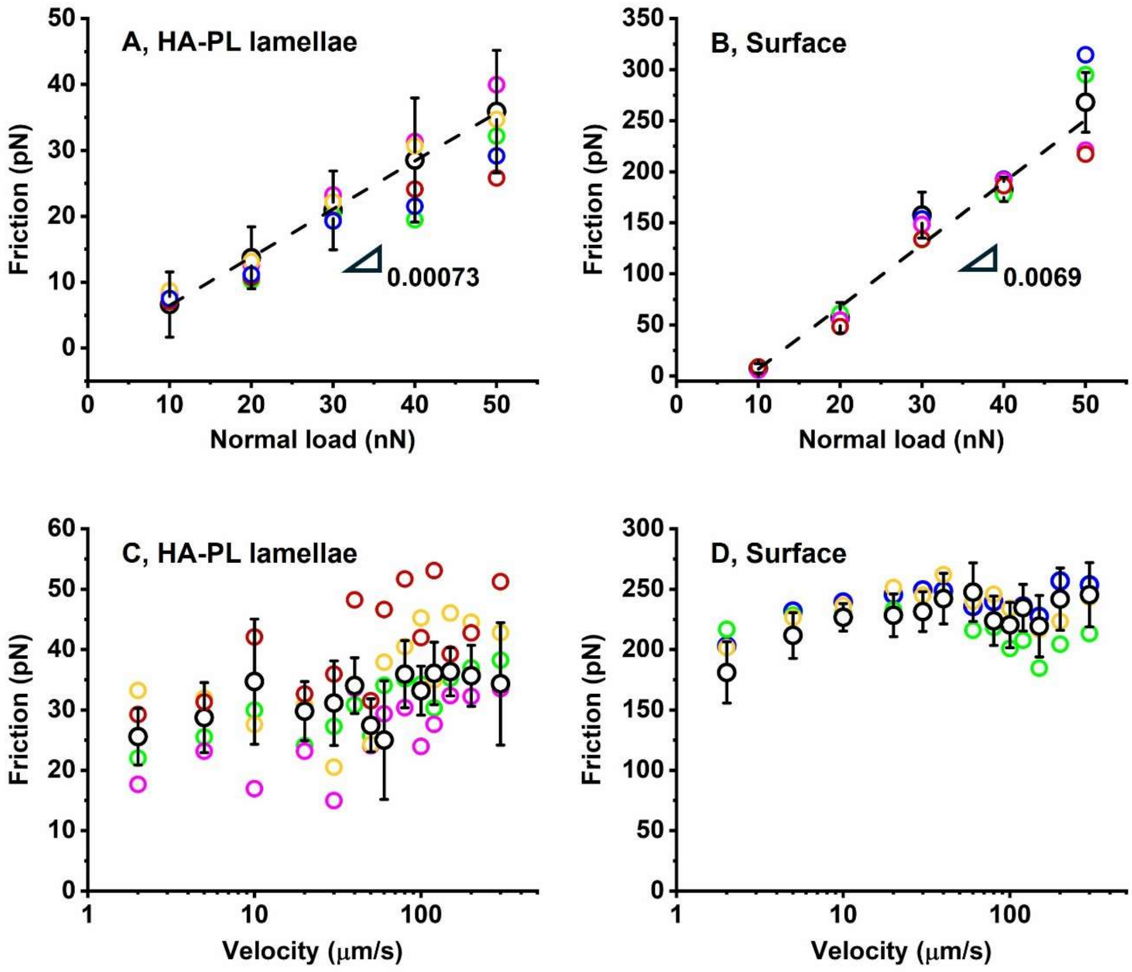

Fig. S14.
Friction measurements on *healthy* HA-PL films. Friction measurements were carried out with a gold colloid. The colloid is coated with the boundary film but the HA-PL sample is *not* present in the liquid, and therefore, self-healing is excluded. Each color represents a spot on the surface: friction vs. load on (**A**) HA-PL lamellae and (**B**) surface, as well as friction vs. velocity on the (**C**) HA-PL lamellae and (**D**) surface. Load-dependent friction measurements were performed at a fixed lateral sliding velocity of 5 μm/s and loads ranging from 10 to 50 nN. Friction vs. velocity was carried out under a constant normal load of 40 nN and sliding velocity ranging from 2 to 300 μm/s. Each data point in the scatter plot represents the average of a friction histogram taken within a region of an image. The error bar represents the standard deviation of each histogram.

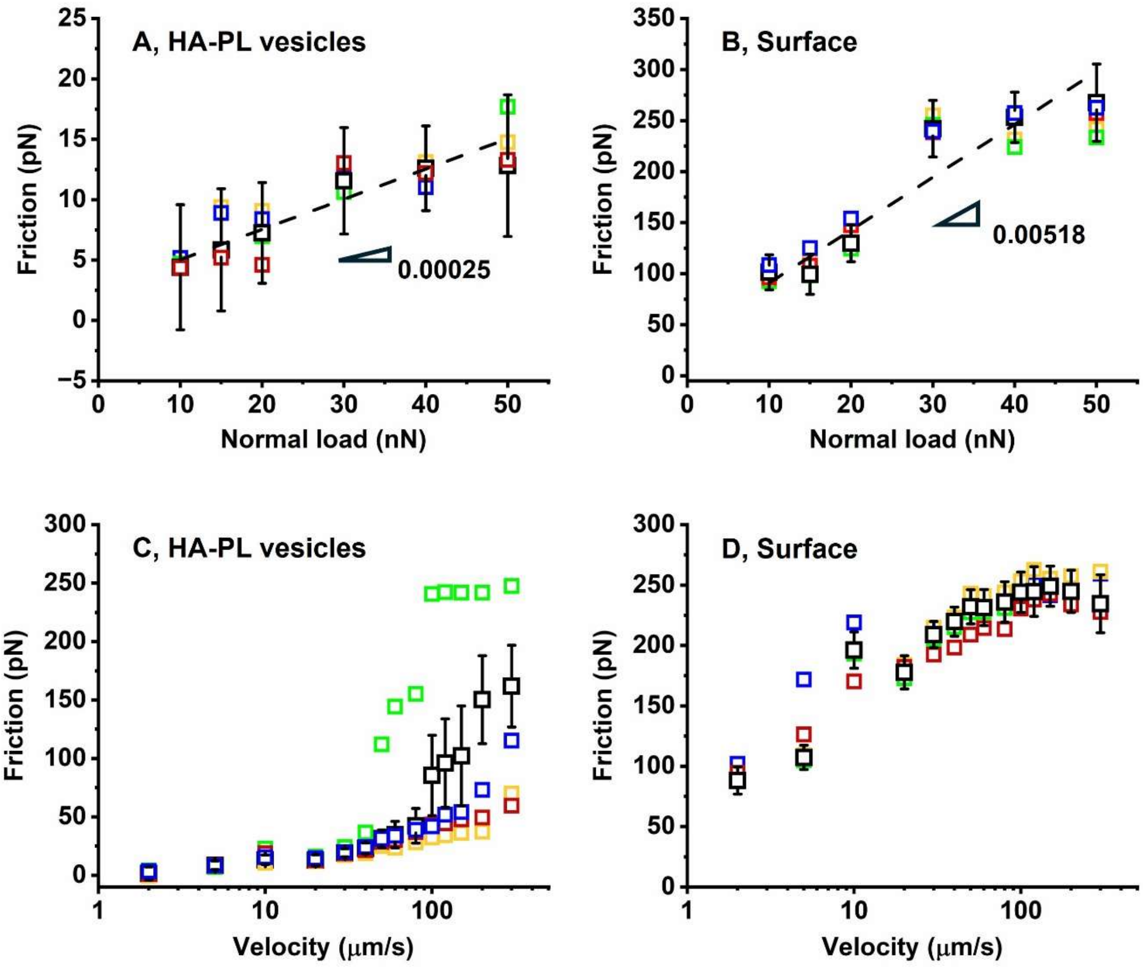

Fig. S15.
Friction measurements on *OA* HA-PL films. Friction measurements were carried out with a gold colloid. The colloid is coated with the boundary film but the HA-PL sample is *not* present in the liquid, and therefore, self-healing is excluded. Each color represents a spot on the surface: friction vs. load on (**A**) HA-PL vesicles and (**B**) surface, as well as friction vs. velocity on the (**C**) HA-PL vesicles and (**D**) surface. Load-dependent friction measurements were performed at a fixed lateral sliding velocity of 5 μm/s and loads ranging from 10 to 50 nN. Friction vs. velocity was varied out under a constant normal load of 40 nN and sliding velocity ranging from 2 to 300 μm/s. Each data point in the scatter plot represents the average of a friction histogram taken within a region of an image. The error bar represents the standard deviation of each histogram.

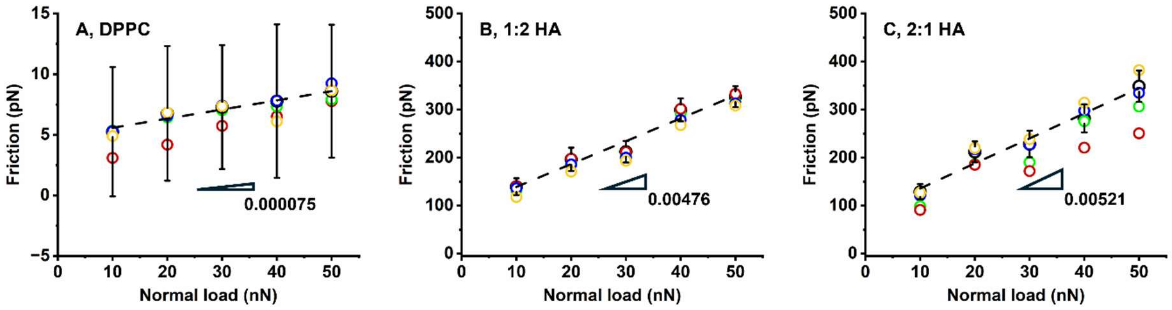

Fig. S16.

Friction measurements as function of load on *reference* samples. The color code represents different locations for (**A**) DPPC vesicles, (**B**)1: 2 HA mixtures, (**C**) 2: 1 HA mixtures. The measurements were conducted with the molecule in the liquid phase. The normal load was varied across a range of 10 – 50 nN and the sliding velocity was maintained constat at 5 µm/s. Each data point in the scatter plot represents the average of a friction histogram taken within a region of an image. The error bar represents the standard deviation of each histogram.

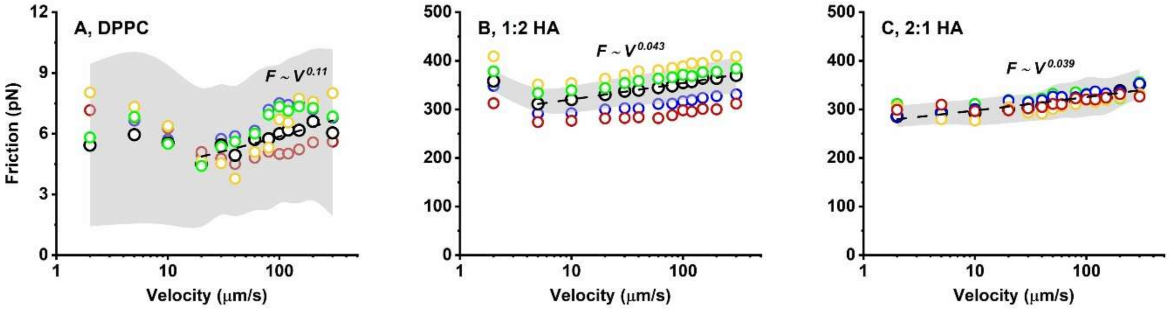

Fig. S17.

Friction measurements as function of velocity on *reference* samples. The color code represents different locations for (**A**) DPPC vesicles, (**B**) 1: 2 HA mixtures, (**C**) 2: 1 HA mixtures. The measurements were conducted with the molecule in the liquid phase. The normal load was maintained constant at 40 nN and the sliding velocity was increased from 2 to 300 μm/s. Each data point in the scatter plot represents the average of a friction histogram taken within a region of an image. The error bar represents the standard deviation of each histogram.

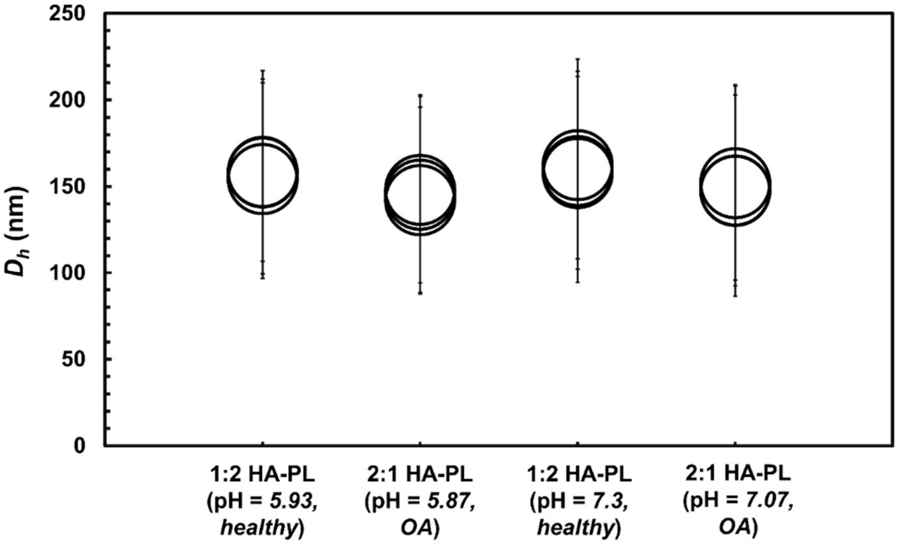

Fig. S18.
Hydrodynamic diameter of HA–PL complex solutions at various pH values. The bubble size represents the relative intensity contribution of each peak, and the error bars indicate the standard deviation of the hydrodynamic diameter across three independent measurements. Under physiological conditions, the synovial fluid in healthy joints maintains a near-neutral pH of about 7.4. However, with the onset and progression of osteoarthritis, metabolic and inflammatory changes lead to acidification of the joint microenvironment, with reported pH values decreasing to 6.6–7.2, and in severe cases approaching 6.0.(*70*) Hyaluronic acid carboxylates ($pK_a \approx 3$) are still >99.8% deprotonated at pH ~ 6(*71*), so the polymer remains highly anionic. A previous study also indicates that the HA molecules properties are stable in a relatively wide pH window ~ 4-11(*72*). The lipid component is DPPC, whose phosphocholine headgroup is zwitterionic and essentially pH-independent between pH 5 and 8; therefore, the bilayer carries ~zero net charge at both pH ~ 6 and physiological pH. In unbuffered 150 mM NaCl (Debye length ~0.8 nm), HA–bilayer interactions are dominated by short-range hydration/steric effects rather than long-range electrostatics, and this supports there is no change of interactions between pH ~6 and 7.4. Nonetheless, to evaluate the pH effect for our simplified system, we studied the size of the HA-PL solutions by adding HEPES, since it will not affect the ionic strength as a zwitterionic buffer(*73*). The measured pH of *healthy* simulated condition changed from 5.93 ± 0.02 to 7.3 ± 0.03 while for the *OA* simulated condition, we intentionally modified the pH to 7.07, which is closer to *OA* affected joint environment. The size distributions of the HA-PL complexes were recorded before and after pH modification. Without HEPES, both healthy (1:2 HA–PL, pH = 5.93) and OA (2:1 HA–PL, pH = 5.87) complexes display unimodal size distributions with mean diameters of 156.9 ± 56 nm and 145.1 ± 55.1 nm, respectively. After adding HEPES buffer and adjusting the pH to physiological values (pH ≈ 7), no significant size change was observed: the average diameters slightly increased to 159.6 ± 58.1 nm for the healthy composition and 149 ± 57.5 nm for the OA composition. Overlapping bubbles correspond to the three replicate measurements for each condition. Based on these results, we do not expect significant changes on the surface structure and lubrication with variation of the pH in the range 6-7.4.

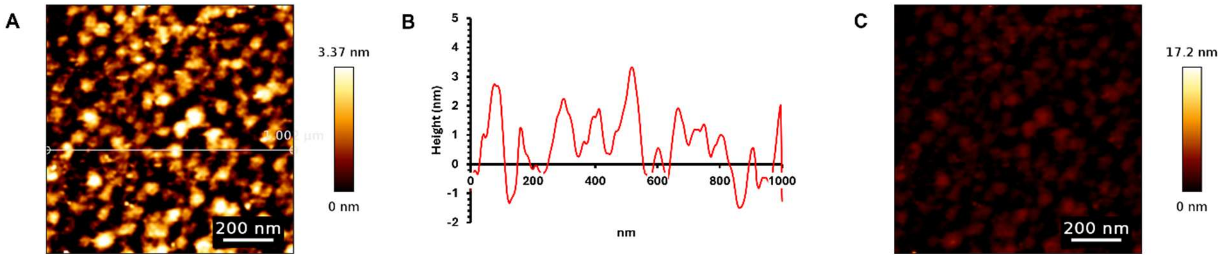

Fig. S19.

Topography images of a clean gold sensor. (A) AFM topography image of a clean bare gold surface. The surface displays nanoscale granular features with a height distribution (<3.4 nm) and RMS roughness of 1.26 nm. The white line indicates the position of the cross-section profile shown in (B). (**B)** Corresponding height profile along the marked line, showing variations in surface topography with peak-to-valley amplitudes of approximately -1.5 - 3 nm over lateral distances of ~1.5 µm. Scan size: 1× 1 µm² as in **Fig. 1A**; scale bar: 200 nm. **(C)** Same topography image as **(A)** but with the same z-scale as **Fig. 1A**. This justifies that the roughness of the gold surface does not affect the interpretation of the topography images with adsorbed HA-PL complexes.

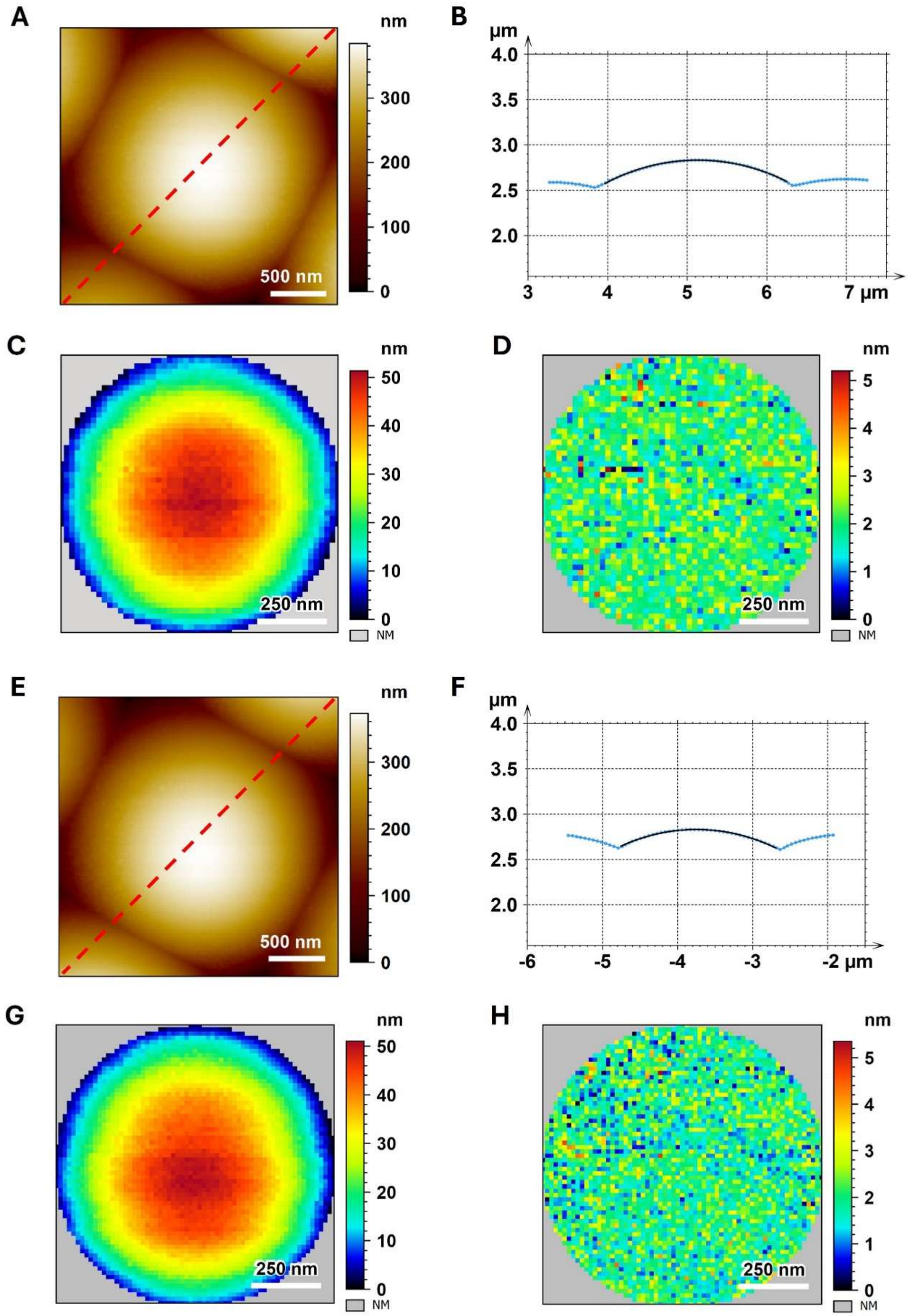

Fig. S20.

Topography of the gold microsphere pre- and post-friction measurements. (A) Reverse image of the gold microsphere and **(B)** representative cross-section height profile to determine

radius, *before* friction measurement. (**C)** Projected area of the center of the hemi-spherical cap and **(D)** surface roughness after removing the curvature of the hemi-spherical surface *before* experiment. **(E)** Reverse image of the gold microsphere. **(F)** Representative cross-section height profile to determine radius, *after* friction measurement. **G)** Projected area of the center of the hemi-spherical cap and **(H)** surface roughness after removing the curvature of the hemi-spherical surface *after* experiment. The radius of curvature was determined by Mountains® software using the 3D images of the microspheres. These images are representative of gold microspheres before and after friction measurements, and the corresponding radii are equal to 2.789 µm and 2.804 µm, respectively. The small difference of ~25 nm between pre- and post-measurement lies within the experimental precision and does not indicate any plastic deformation. If plastic deformation had taken place, we would expect irreversible flattening of the contact area, which was not detected based on these images, confirming that the applied pressures remained well below the yield stress of gold (~205 MPa).

Supplementary Tables

Experiment:	Left	Right	Average
1	51.74	48.55	50.15
2	52.18	48.5	50.34
3	52.24	48.47	50.36
4	52.28	48.42	50.35
5	52.61	48.49	50.55
6	51.18	48.42	49.8
7	51.93	48.41	50.17
8	51.28	48.4	49.84
9	51.06	48.37	49.72
10	51.31	48.41	49.86
Mean:	51.78	48.44	50.11
Std.dev.:	0.17	0.02	0.09

Table S1.
Water contact angle on a clean gold sensor. Ten independent measurements were carried out on both sides of the droplet (**Fig. S2**) to determine the average values and the standard deviation, 50.11°± 0.09.

**Normal load, F (nN)**	**Contact radius, a (nm)**	**Indentation depth, d (pm)**	a/R	d/h	**mean stress $\bar{p}$ (MPa)**
10	7.195	21.8	0.00304	2.19E-04	61.41
15	8.236	28.6	0.00348	2.87E-04	70.30
20	9.065	34.7	0.00383	2.87E-04	77.38
30	10.377	45.4	0.00438	4.55E-04	88.58
40	1..422	55.0	0.00482	5.51E-04	97.49
50	12.304	63.9	0.00519	6.39E-04	105.02

Table S2.
Hertzian contact pressure and parameters for Au–Au contacts in AFM experiments. The calculated pressures overestimate the true contact pressure since they neglect the influence of the adsorbed films. Details of the model and parameters are given in the Methods section.

REFERENCES

1. P. Dantas, S. R. Gonçalves, A. Grenho, V. Mascarenhas, J. Martins, M. Tavares da Silva, S. B. Gonçalves, J. Guimarães Consciência, Hip joint contact pressure and force: A scoping review of in vivo and cadaver studies. *Bone Joint Res.* **12**, 712–721 (2023).

2. I. Clarke, R. Contini, R. Kenedi, Friction and wear studies of articular cartilage: A scanning electron microscope study. *J. Lubr. Technol.* **97**, 358–366 (1975).

3. U. Raviv, J. Klein, Fluidity of bound hydration layers. *Science* **297**, 1540–1543 (2002).

4. A. Dėdinaitė, D. F. Wieland, P. Bełdowski, P. M. Claesson, Biolubrication synergy: Hyaluronan – Phospholipid interactions at interfaces. *Adv. Colloid Interface Sci.* **274**, 102050 (2019).

5. R. Sorkin, N. Kampf, Y. Dror, E. Shimoni, J. Klein, Origins of extreme boundary lubrication by phosphatidylcholine liposomes. *Biomaterials* **34**, 5465–5475 (2013).

6. R. Sorkin, N. Kampf, L. Zhu, J. Klein, Hydration lubrication and shear-induced self-healing of lipid bilayer boundary lubricants in phosphatidylcholine dispersions. *Soft Matter* **12**, 2773–2784 (2016).

7. T. M. Tamer, Hyaluronan and synovial joint: Function, distribution and healing. *Interdiscip. Toxicol.* **6**, 111–125 (2013).

8. M. Wang, C. Liu, E. Thormann, A. Dėdinaitė, Hyaluronan and phospholipid association in biolubrication. *Biomacromolecules* **14**, 4198–4206 (2013).

9. J. Seror, L. Zhu, R. Goldberg, A. J. Day, J. Klein, Supramolecular synergy in the boundary lubrication of synovial joints. *Nat. Commun.* **6**, 6497 (2015).

10. R. Goldberg, A. Schroeder, G. Silbert, K. Turjeman, Y. Barenholz, J. Klein, Boundary lubricants with exceptionally low friction coefficients based on 2D close-packed phosphatidylcholine liposomes. *Adv. Mater.* **23**, 3517–3521 (2011).

11. S. Hollinger, J. M. Georges, D. Mazuyer, G. Lorentz, O. Aguerre-Chariol, N. Du, High-pressure lubrication with lamellar structures in aqueous lubricant. *Tribol. Lett.* **9**, 143–151 (2001).

12. R. Sorkin, Y. Dror, N. Kampf, J. Klein, Mechanical stability and lubrication by phosphatidylcholine boundary layers in the vesicular and in the extended lamellar phases. *Langmuir* **30**, 5005–5014 (2014).

13. A. G. Ogston, J. E. Stanier, The physiological function of hyaluronic acid in synovial fluid; viscous, elastic and lubricant properties. *J. Physiol.* **119**, 244–252 (1953).

14. T. C. Laurent, U. B. Laurent, J. R. Fraser, Functions of hyaluronan. *Ann. Rheum. Dis.* **54**, 429–432 (1995).

15. J. Seror, Y. Merkher, N. Kampf, L. Collinson, A. J. Day, A. Maroudas, J. Klein, Normal and shear interactions between hyaluronan–aggrecan complexes mimicking possible boundary lubricants in articular cartilage in synovial joints. *Biomacromolecules* **13**, 3823–3832 (2012).

16. W. Lin, Z. Liu, N. Kampf, J. Klein, The role of hyaluronic acid in cartilage boundary lubrication. *Cells* **9**, 1606 (2020).

17. M. Benz, N. Chen, G. Jay, J. Israelachvili, Static forces, structure and flow properties of complex fluids in highly confined geometries. *Ann. Biomed. Eng.* **33**, 39–51 (2005).

18. M. Benz, N. Chen, J. Israelachvili, Lubrication and wear properties of grafted polyelectrolytes, hyaluronan and hylan, measured in the surface forces apparatus. *J. Biomed. Mater. Res. A* **71A**, 6–15 (2004).

19. E. Fouissac, M. Milas, M. Rinaudo, Shear-rate, concentration, molecular weight, and temperature viscosity dependences of hyaluronate, a wormlike polyelectrolyte. *Macromolecules* **26**, 6945–6951 (1993).

20. L. Zhu, J. Seror, A. J. Day, N. Kampf, J. Klein, Ultra-low friction between boundary layers of hyaluronan-phosphatidylcholine complexes. *Acta Biomater.* **59**, 283–292 (2017).

21. K. Sun, T. Shoaib, M. W. Rutland, J. Beller, C. Do, R. M. Espinosa-Marzal, Insight into the assembly of lipid-hyaluronan complexes in osteoarthritic conditions. *Biointerphases* **18**, 021005 (2023).

22. Z. Liu, W. Lin, Y. Fan, N. Kampf, Y. Wang, J. Klein, Effects of hyaluronan molecular weight on the lubrication of cartilage-emulating boundary layers. *Biomacromolecules* **21**, 4345–4354 (2020).

23. S. Li, L. Macakova, P. Bełdowski, P. Claesson, A. Dedinaite, Phospholipids and hyaluronan: From molecular interactions to nano- and macroscale friction. *Colloids Interfaces* **6**, 38 (2022).

24. E. H. Miller, Viscosupplementation: therapeutic mechanisms and clinical potential in osteoarthritis of the knee. *J. Am. Acad. Orthop. Surg.* **9**, 146–147 (2001).

25. L. W. Moreland, Intra-articular hyaluronan (hyaluronic acid) and hylans for the treatment of osteoarthritis: Mechanisms of action. *Arthritis Res. Ther.* **5**, 54–67 (2003).

26. L. R. Gale, Y. Chen, B. A. Hills, R. Crawford, Boundary lubrication of joints: Characterization of surface-active phospholipids found on retrieved implants. *Acta Orthop.* **78**, 309–314 (2007).

27. N. Kucerka, S. Tristram-Nagle, J. F. Nagle, Closer look at structure of fully hydrated fluid phase DPPC bilayers. *Biophys. J.* **90**, L83–L85 (2006).

28. B. Bagheri, P. Boonnoy, J. Wong-ekkabut, M. Karttunen, Effect of oxidation on POPC lipid bilayers: Anionic carboxyl group plays a major role. *Phys. Chem. Chem. Phys.* **25**, 18310–18321 (2023).

29. S. Garantziotis, R. C. Savani, Hyaluronan biology: A complex balancing act of structure, function, location and context. *Matrix Biol.* **78-79**, 1–10 (2019).

30. T. C. Laurent, J. R. E. Fraser, Hyaluronan. *FASEB J.* **6**, 2397–2404 (1992).

31. J. Peck, A. Slovek, P. Miro, N. Vij, B. Traube, C. Lee, A. A. Berger, H. Kassem, A. D. Kaye, W. F. Sherman, A. Abd-Elsayed, A comprehensive review of viscosupplementation in osteoarthritis of the knee. *Orthop. Rev* **13**, 25549 (2021).

32. A. Y. Hui, W. J. McCarty, K. Masuda, G. S. Firestein, R. L. Sah, A systems biology approach to synovial joint lubrication in health, injury, and disease. *WIREs Syst. Biol. Med.* **4**, 15–37 (2012).

33. P. Smith, R. M. Ziolek, E. Gazzarrini, D. M. Owen, C. D. Lorenz, On the interaction of hyaluronic acid with synovial fluid lipid membranes. *Phys. Chem. Chem. Phys.* **21**, 9845–9857 (2019).

34. T. Zander, D. C. F. Wieland, A. Raj, M. Wang, B. Nowak, C. Krywka, A. Dėdinaitė, P. M. Claesson, V. M. Garamus, A. Schreyer, R. Willumeit-Römer, The influence of hyaluronan on the structure of a DPPC—Bilayer under high pressures. *Colloids Surf. B Biointerfaces* **142**, 230–238 (2016).

35. Z. Pawlak, A. D. Petelska, W. Urbaniak, K. Q. Yusuf, A. Oloyede, Relationship between wettability and lubrication characteristics of the surfaces of contacting phospholipid-based membranes. *Cell Biochem. Biophys.* **65**, 335–345 (2013).

36. R. Richter, A. Mukhopadhyay, A. Brisson, Pathways of lipid vesicle deposition on solid surfaces: A combined QCM-D and AFM study. *Biophys. J.* **85**, 3035–3047 (2003).

37. C. Hamai, P. S. Cremer, S. M. Musser, Single giant vesicle rupture events reveal multiple mechanisms of glass-supported bilayer formation. *Biophys. J.* **92**, 1988–1999 (2007).

38. V. N. Ngassam, W. C. Su, D. L. Gettel, Y. Deng, Z. Yang, N. Wang-Tomic, V. P. Sharma, S. Purushothaman, A. N. Parikh, Recurrent dynamics of rupture transitions of giant lipid vesicles at solid surfaces. *Biophys. J.* **120**, 586–597 (2021).

39. V. P. Zhdanov, Mechanism of rupture of single adsorbed vesicles. *Chem. Phys. Lett.* **641**, 20–22 (2015).

40. O. Allerbo, A. Lundstrom, K. Dimitrievski, Simulations of lipid vesicle rupture induced by an adjacent supported lipid bilayer patch. *Colloids Surf. B Biointerfaces* **82**, 632–636 (2011).

41. W. A. Ducker, T. J. Senden, R. M. Pashley, Direct measurement of colloidal forces using an atomic force microscope. *Nature* **353**, 239–241 (1991).

42. J. Ralston, I. Larson, M. W. Rutland, A. A. Feiler, M. Kleijn, Atomic force microscopy and direct surface force measurements – (IUPAC technical report). *Pure Appl. Chem.* **77**, 2149–2170 (2005).

43. J. Valderas-Gutiérrez, R. Davtyan, C. N. Prinz, E. Sparr, P. Jönsson, H. Linke, F. Höök, Comparative kinetics of supported lipid bilayer formation on silica coated vertically oriented highly curved nanowires and planar silica surfaces. *Nano Lett.* **25**, 3085–3092 (2025).

44. M. Rinaldin, P. Fonda, L. Giomi, D. J. Kraft, Lipid exchange enhances geometric pinning in multicomponent membranes on patterned substrates. *Soft Matter* **16**, 4932–4940 (2020).

45. D. H. de Jong, A. Heuer, The influence of solid scaffolds on flat and curved lipid membranes. *AIP Adv.* **7**, 075007 (2017).

46. E. D. Bonnevie, D. Galesso, C. Secchieri, I. Cohen, L. J. Bonassar, Elastoviscous transitions of articular cartilage reveal a mechanism of synergy between lubricin and hyaluronic acid. *PLOS ONE* **10**, e0143415 (2015).

47. R. Simič, M. Yetkin, K. Zhang, N. D. Spencer, Importance of hydration and surface structure for friction of acrylamide hydrogels. *Tribol. Lett.* **68**, 64 (2020).

48. Y. Gombert, R. Simič, F. Roncoroni, M. Dübner, T. Geue, N. D. Spencer, Structuring hydrogel surfaces for tribology. *Adv. Mater. Interfaces* **6**, 1901320 (2019).

49. J. Yu, X. Banquy, G. W. Greene, D. D. Lowrey, J. N. Israelachvili, The boundary lubrication of chemically grafted and cross-linked hyaluronic acid in phosphate buffered saline and lipid solutions measured by the surface forces apparatus. *Langmuir* **28**, 2244–2250 (2012).

50. G. D. Jay, Characterization of a bovine synovial fluid lubricating factor. I. Chemical, Surface activity and lubricating properties. *Connect. Tissue Res.* **28**, 71–88 (1992).

51. R. Goldberg, A. Schroeder, Y. Barenholz, J. Klein, Interactions between adsorbed hydrogenated soy phosphatidylcholine (HSPC) vesicles at physiologically high pressures and salt concentrations. *Biophys. J.* **100**, 2403–2411 (2011).

52. S. Jahn, J. Klein, Hydration lubrication: The macromolecular domain. *Macromolecules* **48**, 5059–5075 (2015).

53. S. D. Paolo, M. Wesseling, M. Pastrama, S. Van Rossom, G. Valente, I. Jonkers, Cartilage contact pressure in the knee in the presence of a focal defect. *Orthop. Proc.* **100-B**, 86–86 (2018).

54. S. Salari, A. Beheshti, Asperity-based contact and static friction with provision for creep: A review. *Surf. Interfaces* **24**, 101144 (2021).

55. A. Gaisinskaya-Kipnis, J. Klein, Normal and frictional interactions between liposome-bearing biomacromolecular bilayers. *Biomacromolecules* **17**, 2591–2602 (2016).

56. J.-P. Colletier, B. Chaize, M. Winterhalter, D. Fournier, Protein encapsulation in liposomes: Efficiency depends on interactions between protein and phospholipid bilayer. *BMC Biotechnol.* **2**, 9 (2002).

57. M. V. Voinova, M. Rodahl, M. Jonson, B. Kasemo, Viscoelastic acoustic response of layered polymer films at fluid-solid interfaces: Continuum mechanics approach. *Phys. Scr.* **59**, 391–396 (1999).

58. A. A. Feiler, L. Bergström, M. W. Rutland, Superlubricity using repulsive van der Waals forces. *Langmuir* **24**, 2274–2276 (2008).

59. R. W. Carpick, M. Salmeron, Scratching the surface: Fundamental investigations of tribology with atomic force microscopy. *Chem. Rev.* **97**, 1163–1194 (1997).

60. P. C. Nalam, N. N. Gosvami, M. A. Caporizzo, R. J. Composto, R. W. Carpick, Nano-rheology of hydrogels using direct drive force modulation atomic force microscopy. *Soft Matter* **11**, 8165–8178 (2015).

61. N. Bibissidis, K. Betlem, G. Cordoyiannis, F. P.-v. Bonhorst, J. Goole, J. Raval, M. Daniel, W. Góźdź, A. Iglič, P. Losada-Pérez, Correlation between adhesion strength and phase behaviour in solid-supported lipid membranes. *J. Mol. Liq.* **320**, 114492 (2020).

62. G. Cordoyiannis, L. Bar, P. Losada-Pérez,“Recent advances in quartz crystal microbalance with dissipation monitoring: Phase transitions as descriptors for specific lipid membrane studies,” in *Adv. Biomembr. Lipid Self-Assem*. (Academic London, 2021), vol. 34, pp. 107–128.

63. D. Drabik, G. Chodaczek, S. Kraszewski, M. Langner, Mechanical properties determination of DMPC, DPPC, DSPC, and HSPC solid-ordered bilayers. *Langmuir* **36**, 3826–3835 (2020).

64. D. Pinisetty, D. Moldovan, R. Devireddy, The effect of methanol on lipid bilayers: An atomistic investigation. *Ann. Biomed. Eng.* **34**, 1442–1451 (2006).

65. Q. Yin, X. Shi, H. Ding, X. Dai, G. Wan, Y. Qiao, Interactions of borneol with DPPC phospholipid membranes: A molecular dynamics simulation study. *Int. J. Mol. Sci.* **15**, 20365–20381 (2014).

66. D. Johannsmann, *The Quartz Crystal Microbalance in Soft Matter Research: Fundamentals and Modeling*. (Springer, Cham, 2015).

67. K. K. Kanazawa, J. G. Gordon, Frequency of a quartz microbalance in contact with liquid. *Anal. Chem.* **57**, 1770–1771 (1985).

68. I. Reviakine, D. Johannsmann, R. P. Richter, Hearing what you cannot see and visualizing what you hear: Interpreting quartz crystal microbalance data from solvated interfaces. *Anal. Chem.* **83**, 8838–8848 (2011).

69. A. Benedetto, F. Heinrich, M. A. Gonzalez, G. Fragneto, E. Watkins, P. Ballone, Structure and stability of phospholipid bilayers hydrated by a room-temperature ionic liquid/water solution: A neutron reflectometry study. *J. Phys. Chem. B* **118**, 12192–12206 (2014).

70. S. Liao, S. Jia, Y. Yue, H. Zeng, J. Lin, P. Liu, Advancements in pH-Responsive nanoparticles for osteoarthritis treatment: Opportunities and challenges. *Front. Bioeng. Biotechnol.* **12**, 1426794 (2024).

71. C. D. Blundell, P. L. DeAngelis, A. Almond, Hyaluronan: The absence of amide–carboxylate hydrogen bonds and the chain conformation in aqueous solution are incompatible with stable secondary and tertiary structure models. *Biochem. J.* **396**, 487–498 (2006).

72. A. Maleki, A.-L. Kjøniksen, B. Nyström, Effect of pH on the behavior of hyaluronic acid in dilute and semidilute aqueous solutions. *Macromol. Symp.* **274**, 131–140 (2008).

73. E. Stellwagen, J. D. Prantner, N. C. Stellwagen, Do zwitterions contribute to the ionic strength of a solution? *Anal. Biochem.* **373**, 407–409 (2008).
